# Supplementary material for: Small RNA‐binding protein RapZ mediates cell envelope precursor sensing and signaling in Escherichia coli
Source: EMBO J. 2020 Feb 17;39(6):e103848. doi: 10.15252/embj.2019103848 (PMC7073468; doi:10.15252/embj.2019103848)
Supplement: Supplementary file 1 — Appendix [file EMBJ-39-e103848-s001.pdf]

# APPENDIX for

# Small RNA-binding protein RapZ mediates cell envelope precursor sensing and signaling in *Escherichia coli*

Muna A. Khan[1], Svetlana Durica-Mitic[1], Yvonne Göpel[1], Ralf Heermann[2] and Boris Görke[1*]

[1]Department of Microbiology, Immunobiology and Genetics, Max Perutz Labs, University of Vienna, Vienna Biocenter (VBC), 1030 Vienna, Austria

[2]Microbiology and Wine Research, Institute for Molecular Physiology, Johannes Gutenberg-University Mainz, 55128 Mainz, Germany.

Running title: A multifunctional sRNA-binding protein

[*]Corresponding author

E-mail: boris.goerke@univie.ac.at

**This Appendix contains:**

Appendix Figures S1 – S15

Appendix Tables S1 – S3

Appendix Supplementary Materials and Methods

Appendix Supplementary References

## TABLE OF CONTENTS

**Appendix Figure S1** Growth curves for the experiment presented in Fig 1A

**Appendix Figure S2** GlcN6P has no impact on GlmY, GlmZ and *glmS* in the absence of RapZ

**Appendix Figure S3** Identification of GlcN6P in the eluate derived from purification of Strep-RapZ under GlcN6P replete conditions

**Appendix Figure S4** Purification profile of Strep-KdpE under GlcN6P replete and depletion conditions

**Appendix Figure S5** GlcN6P depletion upregulates *glmY* expression

**Appendix Figure S6** RapZ has no global role for transcription of $\sigma^{54}$-controlled genes

**Appendix Figure S7** BACTH assay addressing interaction of RapZ with QseF and QseE in the BACTH reporter strain BTH101 containing (*A*) or lacking (*B*) the endogenous *qseEGF* operon

**Appendix Figure S8** Purification profile for QseF-$His_{10}$, QseE'-$His_{10}$ and Strep-RapZ

**Appendix Figure S9** Analysis of the *in vitro* transcribed sRNAs used in Fig 4C to verify RNA integrity

**Appendix Figure 10** GlmY* half-life increases under GlcN6P starvation conditions and RapZ is required for this effect

**Appendix Figure 11** Northern blot experiment addressing half-life of GlmZ in the samples analyzed in Fig 6A

**Appendix Figure 12** Stabilization of GlmY* under GlcN6P starvation conditions is reversible by supplying an exogenous amino sugar

**Appendix Figure 13** Effect of GlcN6P depletion in strains lacking *glmZ* (*A*) or *glmY* (*B*)

**Appendix Figure 14** EMSA experiments demonstrating that GlcN6P has no role for GlmY*/RapZ complexes at alkaline pH

**Appendix Figure 15** Effect of GlcN6P on GlmY*/RapZ interaction at pH 7.0

**Appendix Table S1** Strains used in this study

**Appendix Table S2** Plasmids used in this study

**Appendix Table S3** Oligonucleotides used in this study

**Appendix Supplementary Materials and Methods**

**Appendix Supplementary References**

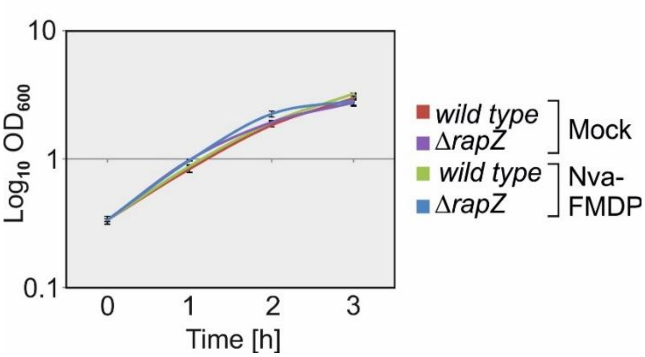


**Appendix Figure S1.** Growth curves for the experiment presented in Fig 1A.

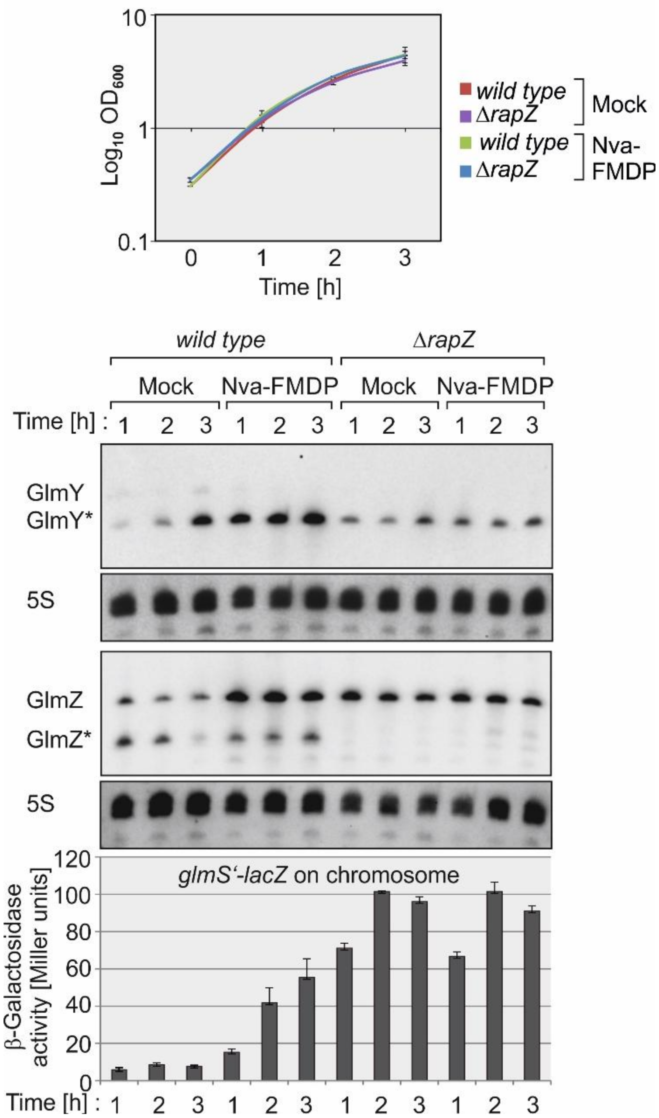


**Appendix Figure S2.** GlcN6P has no impact on GlmY, GlmZ and *glmS* in the absence of RapZ. Similar experiment as shown in Fig 1A, but the MG1655 derivative strains Z854 (*wild type*) and Z857 (Δ*rapZ*) were used. Data information: β-galactosidase activities are presented as mean ± SD; *n* = 2.

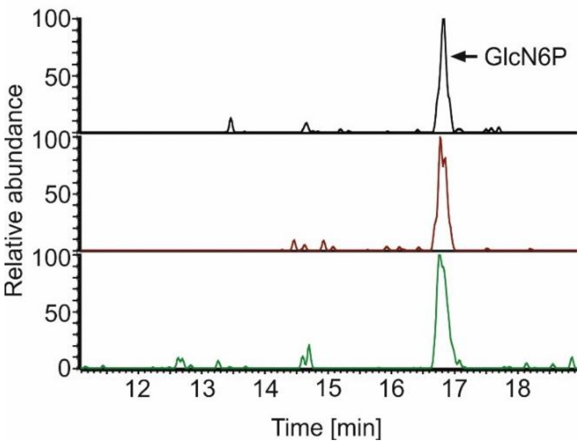


**Appendix Figure S3.** Identification of GlcN6P in the eluate derived from purification of Strep-RapZ under GlcN6P replete conditions. Schematic representation of the MS signals obtained for the metabolite extract prepared from the Strep-RapZ protein eluate. The identity of the detected metabolite was confirmed by analyzing the transitions m/z 258.1 to m/z 199 (black), m/z 258.1 to m/z 79 (red) and m/z 258.1 to m/z 97 (green) and by adding a chemically pure standard of GlcN6P to the sample (Fig 1B, panel v).

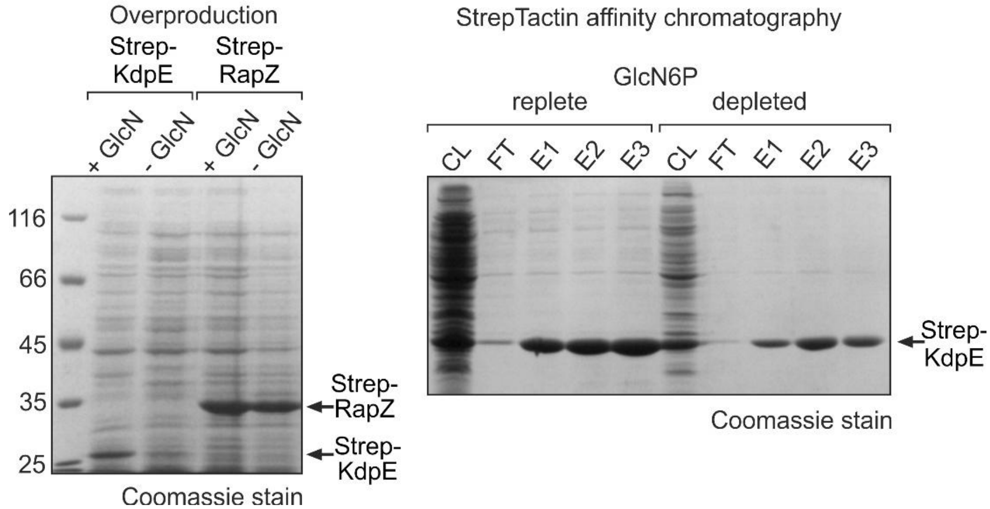


**Appendix Figure S4.** Purification profile of Strep-KdpE under GlcN6P replete and depletion conditions. Left panel: Verification of Strep-KdpE (lanes 1, 2; encoded on plasmid pMK13) and Strep-RapZ (lanes 3, 4) overproduction, respectively, in the $\Delta$*glmS* strain Z904 under GlcN6P replete (+GlcN) and GlcN6P depleted (-GlcN) conditions. The transformants were subsequently used for purification of the respective proteins (right panel for Strep-KdpE and Fig 1B for Strep-RapZ). Right panel: Purification of Strep-KdpE under GlcN6P replete and depleted conditions as described for Strep-RapZ in Fig 1B. The elution fractions E2 were subjected to HILIC-MS/MS.

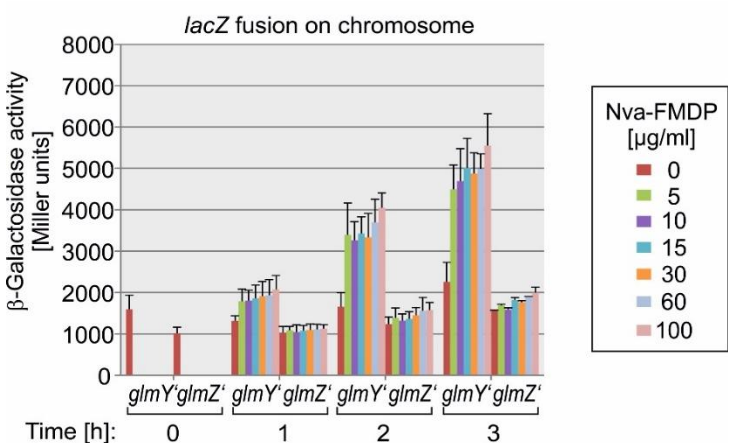


**Appendix Figure S5.** GlcN6P depletion upregulates *glmY* expression. Similar experiment as shown in Fig 2A, but the MG1655 derivative strains Z741 (*glmY'-lacZ*) and Z914 (*glmZ'-lacZ*) were used. Data information: β-galactosidase activities are presented as mean ± SD; $n = 3$.

**Appendix Figure S6**

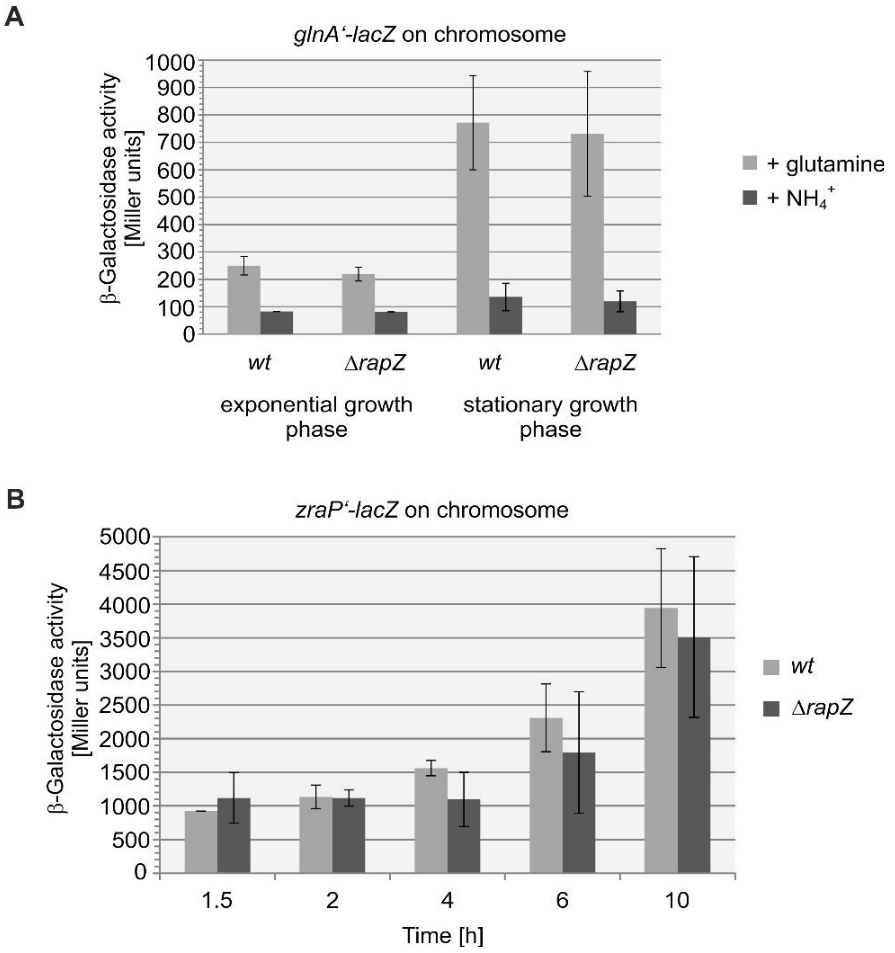


**Appendix Figure S6.** RapZ has no global role for transcription of $\sigma^{54}$-controlled genes. (*A*) Impact of a *ΔrapZ* mutation on expression of a *glnA'-lacZ* reporter fusion under nitrogen replete (+NH4$^{+}$) and nitrogen starvation (+ glutamine) conditions. Strains Z849 (*wild type*) and Z850 (*ΔrapZ*), both of which carry a *glnA'-lacZ* reporter fusion on the chromosome, were grown in MOPS medium ($NH_4^+$, nitrogen replete; (Neidhardt *et al.* 1974)) or in a modified MOPS medium in which $NH_4^+$ was replaced by glutamine. The latter conditions are known to activate the $\sigma^{54}$-dependent $P_{glnA2}$ promoter through NtrB/NtrC (Magasanik 1993). β-Galactosidase activities were determined from exponentially growing and stationary phase grown cells. (*B*) Expression of a chromosomal *zraP'-lacZ* fusion in *wild type* (Z274) and *ΔrapZ* (Z275) strains. Strains were grown in LB supplemented with 1 mM $ZnCl_2$ for induction of *zraP* expression and samples were harvested during growth for determination of β-galactosidase activities. Data information: In (A) and (B) β-galactosidase activities are presented as mean ± SD; *n* = 2.

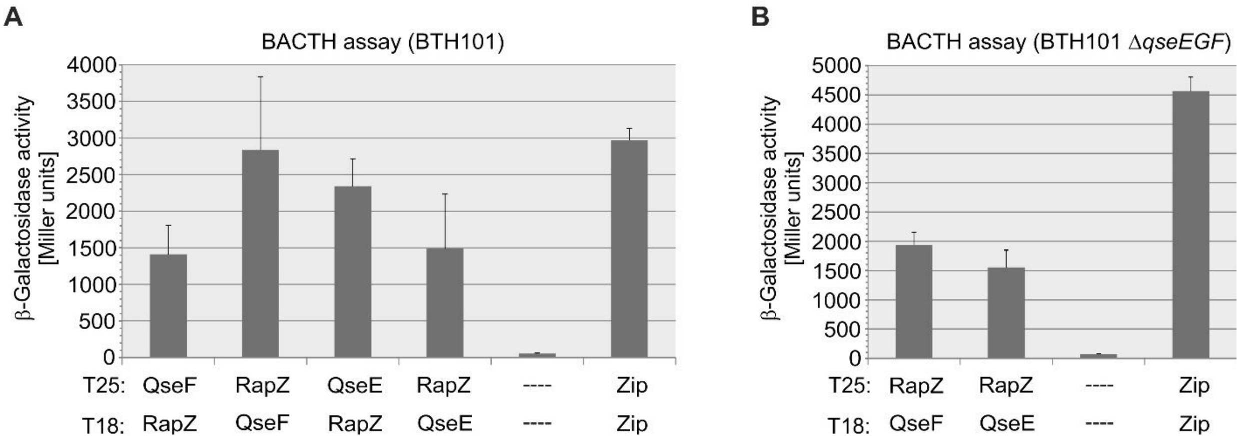


**Appendix Figure S7.** BACTH assay addressing interaction of RapZ with QseF and QseE in the BACTH reporter strain BTH101 containing (*A*) or lacking (*B*) the endogenous *qseEGF* operon. (*A*) Strain BTH101 was used, which harbored the following plasmid combinations: pBGG352 + pBGG349 (column 1), pBGG353 + pBGG348 (column 2), pYG199 + pBGG349 (column 3), pYG246 + pBGG348 (column 4), pKT25 + pUT18C (column 5), pKT25-zip + pUT18C-zip (column 6). (*B*) Strain Z1087 was addressed, which carried the following plasmids: pBGG353 + pBGG348 (column 1), pYG246 + pBGG348 (column 2), pKT25 + pUT18C (column 3), pKT25-zip + pUT18C-zip (column 4). β-Galactosidase activities were determined from cells grown to stationary phase. Data information: In (A) and (B) β-galactosidase activities are presented as mean ± SD; *n* = 3.

## Appendix Figure S8

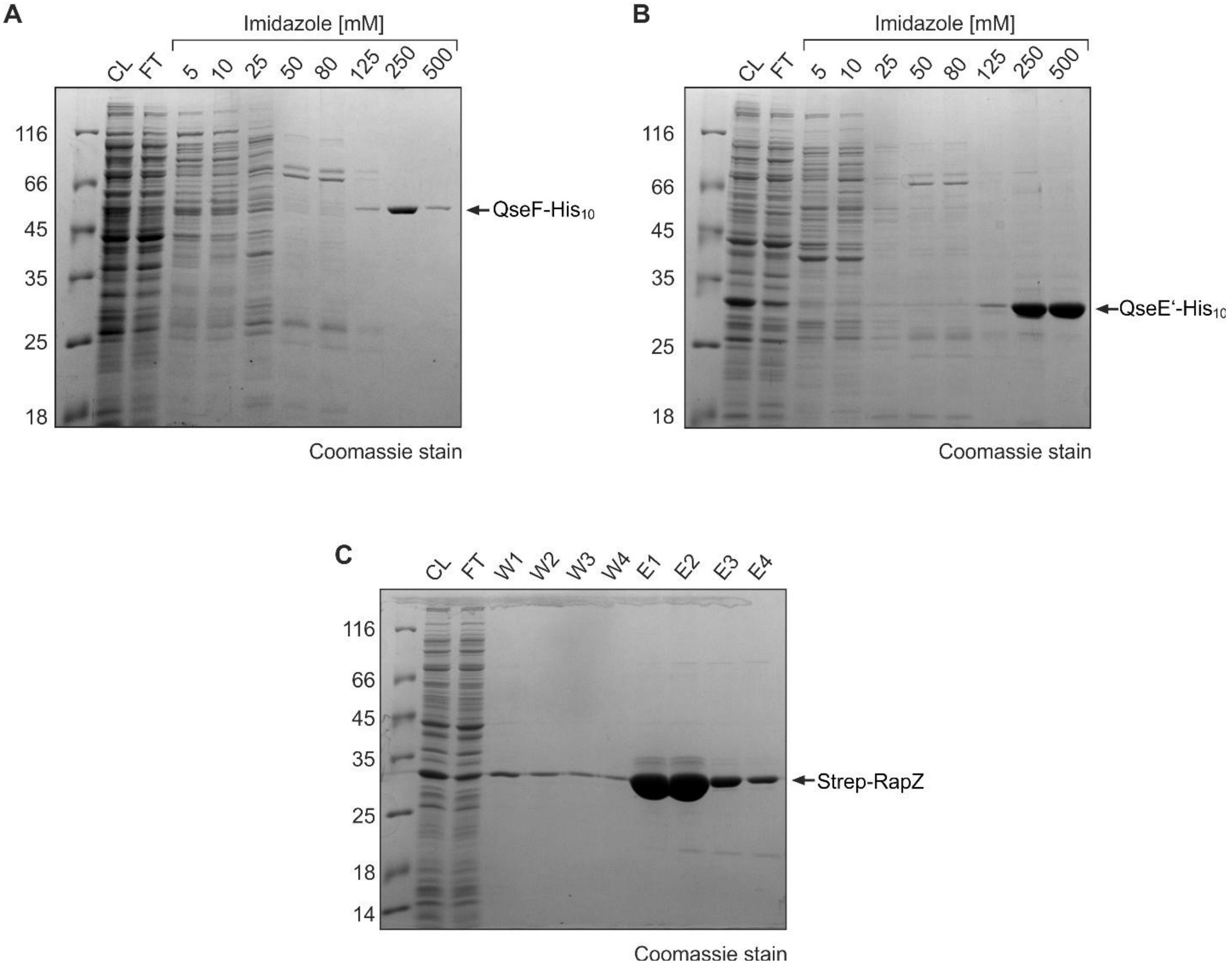


**Appendix Figure S8.** Purification profile for QseF-His$_{10}$, QseE'-His$_{10}$ and Strep-RapZ. Overproduction and purification of (*A*) QseF-His$_{10}$, (*B*) QseE'-His$_{10}$, and (*C*) Strep-RapZ, respectively, as described under "Appendix Supplementary Materials and Methods".

**Appendix Figure S9**

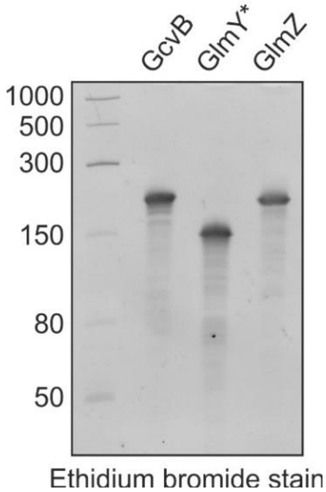


**Appendix Figure S9.** Analysis of the *in vitro* transcribed sRNAs used in Fig 4C to verify RNA integrity. The sRNAs GcvB (205 nt), processed GlmY (GlmY*; 148 nt) and GlmZ (207 nt) were generated by *in vitro* transcription using 40 U T7 RNA polymerase (NEB), 20 U RiboLock RNase inhibitor (Thermo Scientific) and 0.5 mM NTPs (Roche Diagnostics). The templates for the corresponding *in vitro* transcription reactions were obtained by PCR using oligonucleotides BG1792/BG1793 (GcvB), BG446/BG448 (GlmY*) and BG444/BG445 (GlmZ), respectively. The reactions were separated on a denaturing PAA gel (8% PAA, 7 M urea, 1 × TBE) and the sRNA species were excised. Following a phenol:chloroform:isoamyl alcohol (25:24:1) extraction, the sRNAs were precipitated using ethanol:4M lithium chloride (30:1) at -80°C. The pellet was dissolved in 20 µl $H_2 0$ and the RNA concentration was determined using Nanodrop (Thermo Scientific). To check RNA integrity, 500 ng of each sRNA was separated on a denaturing 8% PAA gel, which was subsequently stained with ethidium bromide. An RNA size ladder (NEB) was separated alongside (left).

**Appendix Figure S10**

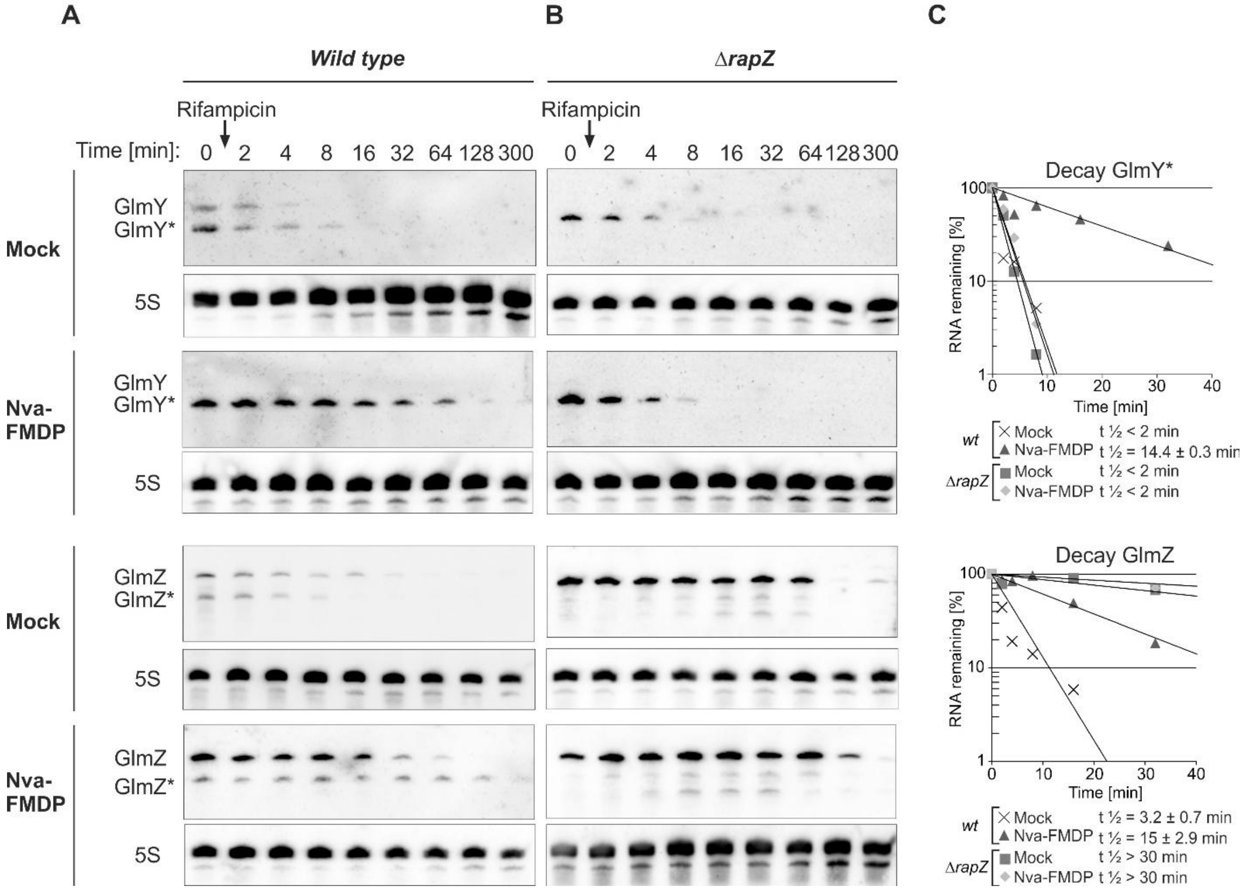


**Appendix Figure S10.** GlmY* half-life increases under GlcN6P starvation conditions and RapZ is required for this effect. Similar experiment as shown in Fig 5, but the MG1655 derivative strains Z854 (*wild type*) and Z857 (Δ*rapZ*) were used. Data information: In (C) data are presented as mean; n = 2. Half-lives are presented as mean ± SD.

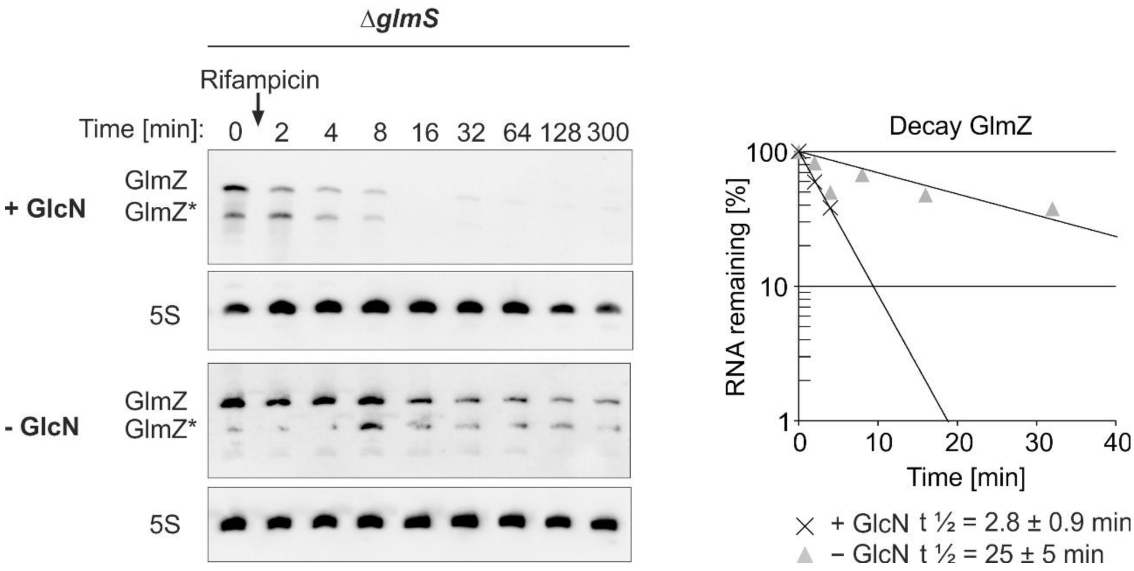


**Appendix Figure S11.** Northern blot experiment addressing half-life of GlmZ in the samples analyzed in Fig 6A. Data information: In the semi-logarithmic plot data are presented as mean; n = 2. Half-lives are presented as mean ± SD.

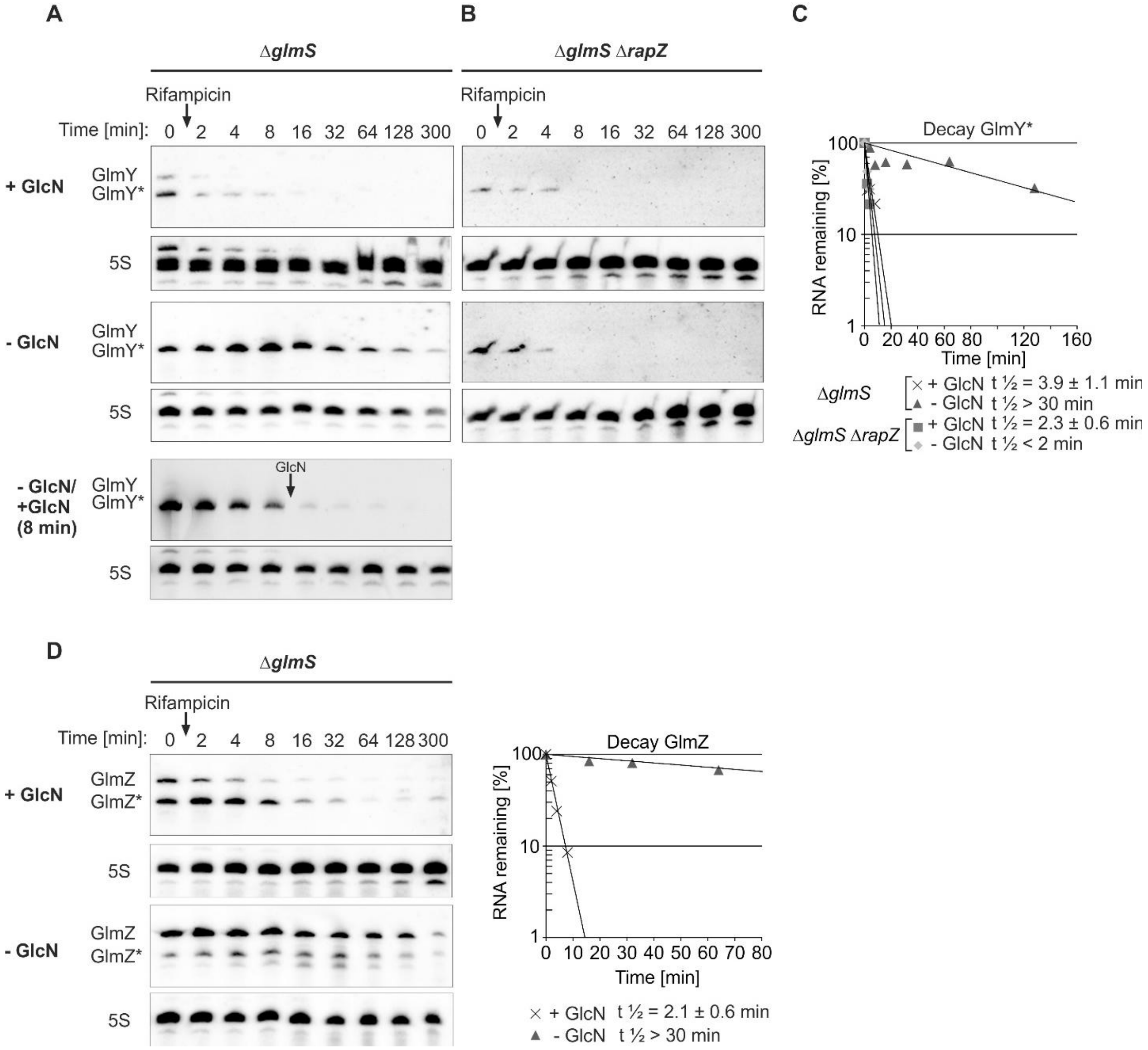


**Appendix Figure S12.** Stabilization of GlmY* under GlcN6P starvation conditions is reversible by supplying an exogenous amino sugar. (*A*) Similar experiment as shown in Fig 6A but the MG1655 derivative strain Z904 was used. (*B*) Similar experiment as shown in Fig 6B but the MG1655 derivative strain Z939 was used. (*C*) Semi-logarithmic plots of GlmY* decay for half-life determination. (*D*) Similar experiment as shown in Appendix Figure S11 but the MG1655 derivative strain Z904 was addressed. The samples used in Appendix Figure S12A were analyzed using a probe directed against GlmZ. Data information: In the semi-logarithmic plots in (C) and (D) data are presented as mean; n = 2. Half-lives are presented as mean ± SD.

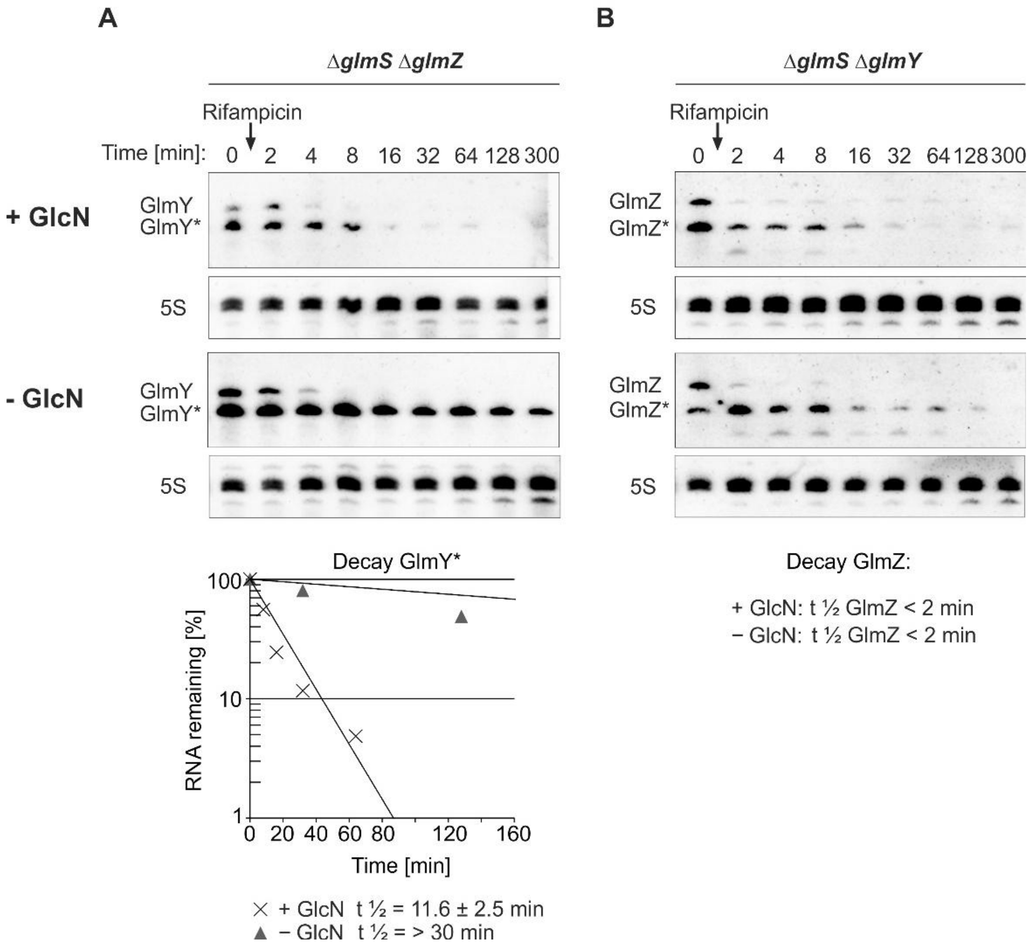


**Appendix Figure S13.** Effect of GlcN6P depletion in strains lacking *glmZ* (*A*) or *glmY* (*B*). Strains Z932 (*ΔglmS ΔglmZ*; (*A*)) and Z931 (*ΔglmS ΔglmY*; (*B*)) were grown in presence or absence of 0.2% GlcN and decay of GlmY* (*A*) and GlmZ (*B*) was addressed as described for Fig 6. Data information: In the semi-logarithmic plots in (A) data are presented as mean; n = 2. Half-lives are presented as mean ± SD.

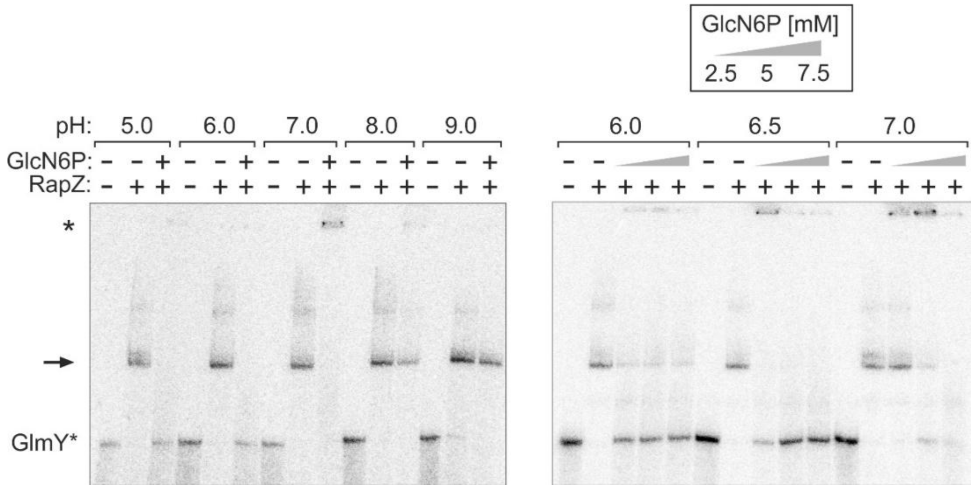


**Appendix Figure S14.** EMSA experiments demonstrating that GlcN6P has no role for GlmY*/RapZ complexes at alkaline pH. Left panel: $\alpha$-$^{32}$P-UTP labelled GlmY* was incubated with 1200 nM RapZ in the absence or presence of 7.5 mM GlcN6P as indicated. The pH of the binding buffer was adjusted to the indicated value. Right panel: $\alpha$-$^{32}$P-UTP labelled GlmY* was incubated with 1200 nM RapZ in the absence or presence of three different GlcN6P concentrations (2.5 mM, 5 mM and 7.5 mM). The pH of the binding buffer was adjusted to the indicated value.

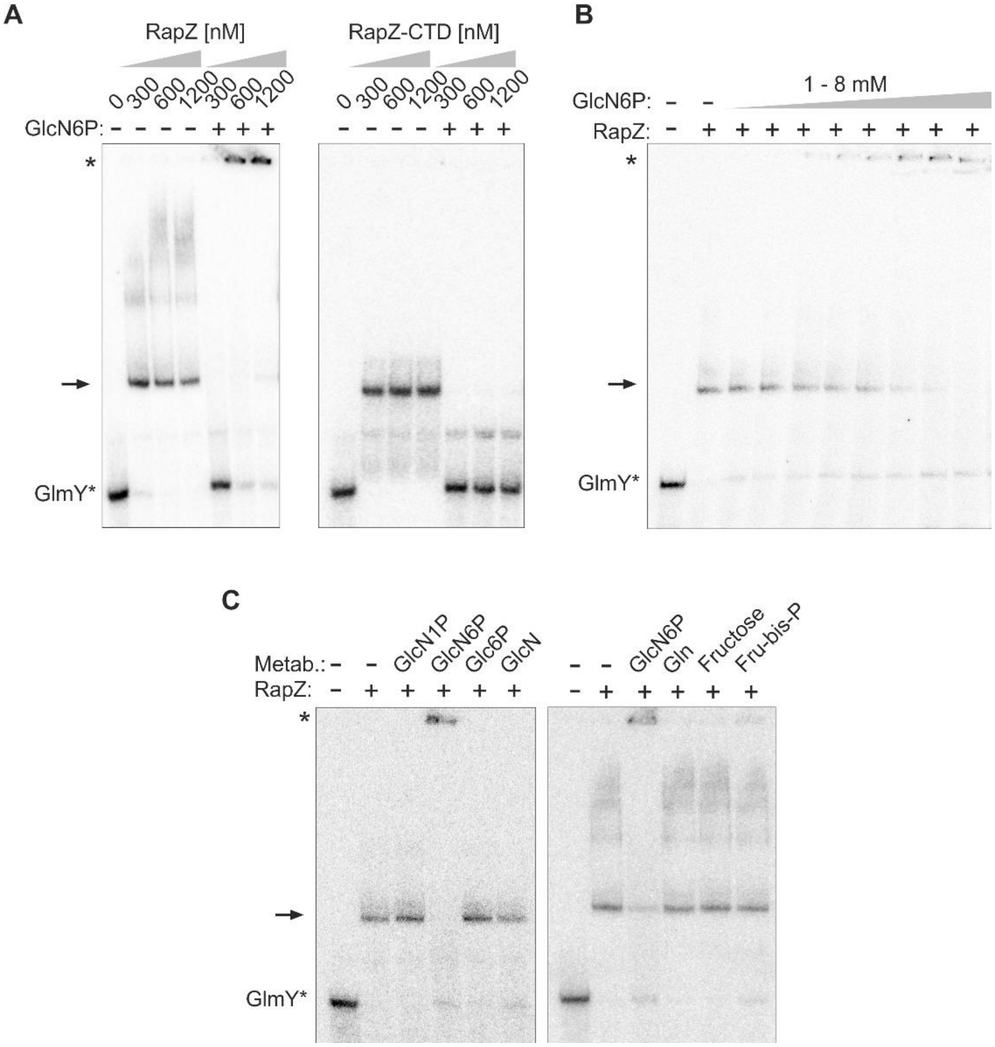


**Appendix Figure S15.** Effect of GlcN6P on GlmY*/RapZ interaction at pH 7.0. Similar experiments as shown in Fig 6D-F, but as a difference, the pH of the binding buffer was adjusted to 7.0.

# Appendix Tables

## **Appendix Table S1**. Strains used in this study

| Name | Genotype | Reference |
|---|---|---|
| BTH101 | *$F^-$ cya-99 araD139 galE15 galK16 rpsL1 ($Str^R$) hsdR2 mcrA1 mcrB1* | (Karimova *et al.* 1998) |
| IBPC750 | *thi-1, argG6, argE3, his-4, xyl-5, rpsL, ΔlacX74, mlc, ΔglmS ::tet* | (Plumbridge & Vimr 1999) |
| JW2538 | *Δ(araD-araB)567 ΔlacZ4787(::rrnB-3) $\lambda^-$ ΔqseF728::kan rph-1 Δ(rhaD-rhaB)568 hsdR514* | (Baba *et al.* 2006) |
| JW5407 | *Δ(araD-araB)567 ΔlacZ4787(::rrnB-3) $\lambda^-$ ΔqseE730::kan rph-1 Δ(rhaD-rhaB)568 hsdR514* | (Baba *et al.* 2006) |
| R1279 | CSH50 *Δ*(*pho-bgl*)201 *Δ*(*lac-pro*) *ara thi* | (Schnetz *et al.* 1996) |
| RH785 | MG1655 *ΔcyaA* | (Abel *et al.* 2011) |
| S4197 | MG1655 *$rph^+$ $ilvG^+$ ΔlacZ* | (Venkatesh *et al.* 2010) |
| Z8 | R1279 *λattB::*[*aadA, glmS'-lacZ*], *$strp^R$, F'($lacI^q$)* | (Kalamorz *et al.* 2007) |
| Z24 | R1279 *ΔrapZ::cat* | (Kalamorz *et al.* 2007) |
| Z28 | R1279 *ΔrapZ λattB::*[*aadA, glmS'-lacZ*], *$strp^R$* , *F'*(*$lacI^q$*) | (Kalamorz *et al.* 2007) |
| Z37 | R1279 *ΔrapZ* | (Kalamorz *et al.* 2007) |
| Z106 | R1279 *ΔglmY ΔglmZ* | (Göpel *et al.* 2013) |
| Z190 | R1279 *λattB::*[*aadA*, *glmY'-lacZ,* -10 mutated] | (Reichenbach *et al.* 2009) |
| Z196 | R1279 *ΔqseF λattB::*[*aadA*, *glmY'-lacZ,* -10 mutated] | (Reichenbach *et al.* 2009) |
| Z197 | R1279 *λattB::*[*aadA*, *glmY'-lacZ*] | (Reichenbach *et al.* 2009) |
| Z201 | R1279 *λattB::*[*aadA*, *glmY'-lacZ,* -24 mutated] | (Reichenbach *et al.* 2009) |
| Z225 | R1279 *ΔrapZ λattB::*[*aadA*, *glmY'-lacZ*] | pBGG201/BamHI→Z37; this work |
| Z274 | R1279 *λattB::*[*aadA*, *zraP'-lacZ*] | (Reichenbach *et al.* 2009) |
| Z275 | R1279 *ΔrapZ λattB::*[*aadA*, *zraP'-lacZ*] | pBGG324/BamHI→Z37; this work |
| Z360 | R1279 *λattB::*[*aadA*, *glmZ'-lacZ*] | (Göpel *et al.* 2011) |
| Z741 | S4197 *λattB::*[*aadA*, *glmY'-lacZ*] | pBGG201/BamHI→S4197; this work |
| Z849 | R1279 *λattB::*[*aadA*, *glnA'-lacZ*] | pDL43/BamHI→R1279; this work |
| Z850 | R1279 *ΔrapZ λattB::*[*aadA*, *glnA'-lacZ*] | pDL43/BamHI→Z37; this work |
| Z854 | S4197 *λattB*::[*aadA*, *glmS* -5'::*lacZ*] | (Khan *et al.* 2016) |
| Z855 | S4197 *ΔglmY λattB*::[*aadA*, *glmS* -5'::*lacZ*] | (Khan *et al.* 2016) |
| Z856 | S4197 *ΔglmZ λattB*::[*aadA*, *glmS* -5'::*lacZ*] | (Khan *et al.* 2016) |
| Z857 | S4197 *ΔrapZ λattB*::[*aadA*, *glmS* -5'::*lacZ*] | Z868 cured from *cat*; this work |
| Z868 | S4197 *ΔrapZ::cat λattB*::[*aadA*, *glmS* -5'::*lacZ*] | T4GT7 (Z24) → Z854, this work |
| Z904 | S4197 *ΔglmS::tet λattB*::[*aadA*, *glmS* -5'::*lacZ*] | T4GT7 (IBPC750) → Z854, this work |
| Z914 | S4197 *λattB::*[*aadA*, *glmZ'*(-424 to +32)-*lacZ*] | T4GT7 (Z360) → S4197, this work |
| Z931 | S4197 *ΔglmY ΔglmS::tet λattB*::[*aadA*, *glmS* -5'::*lacZ*] | T4GT7 (IBPC750) → Z855, this work |
| Z932 | S4197 *ΔglmZ ΔglmS::tet λattB*::[*aadA*, *glmS* -5'::*lacZ*] | T4GT7 (IBPC750) → Z856, this work |
| Z939 | S4197 *ΔglmS::tet ΔrapZ λattB*::[*aadA*, *glmS* -5'::*lacZ*] | T4GT7 (IBPC750) → Z857, this work |
| Z967 | R1279 Δ[*qseE*, *qseG*, *qseF*]::*cat* | PCR BG1390+BG1301→R1279, this work |
| Z1067 | R1279 *ΔqseF ΔrapZ::cat λattB::*[*aadA*, *glmY'-lacZ,* -10 mutated] | T4GT7 (Z24) → Z196, this work |
| Z1078 | S4197 *ΔqseF::kan λattB*::[*aadA*, *glmS* -5'::*lacZ*] | T4GT7 (JW2538) → Z854, this work |
| Z1079 | S4197 *ΔqseE::kan λattB*::[*aadA*, *glmS* -5'::*lacZ*] | T4GT7 (JW5407) → Z854, this work |
| Z1080 | S4197 *ΔqseE::kan ΔrapZ λattB*::[*aadA*, *glmS* -5'::*lacZ*] | T4GT7 (JW2538) → Z857, this work |
| Z1081 | S4197 *ΔqseF λattB*::[*aadA*, *glmS* -5'::*lacZ*] | Z1078 cured from *kan*; this work |
| Z1082 | S4197 *ΔqseE λattB*::[*aadA*, *glmS* -5'::*lacZ*] | Z1079 cured from *kan*; this work |
| Z1083 | S4197 *ΔqseF ΔrapZ λattB*::[*aadA*, *glmS* -5'::*lacZ*] | Z1080 cured from *kan*; this work |
| Z1087 | BTH101 Δ[*qseE*, *qseG*, *qseF*]::*cat* | T4GT7 (Z967) → BTH101, this work |
| Z1110 | R1279 *ΔqseF ΔrapZ λattB::*[*aadA*, *glmY'-lacZ,* -10 mutated] | Z1067 cured from *cat*; this work |
| Z1118 | R1279 *ΔglmY ΔglmZ λattB::*[*aadA*, *glmY'-lacZ*] | T4GT7 (Z197) → Z106, this work |
| Z1126 | R1279 *ΔglmS::tet λattB::*[*aadA, glmS'-lacZ*], *$strp^R$, F'*(*$lacI^q$*) | T4GT7 (IBPC750) → Z8, this work |
| Z1127 | R1279 *ΔglmS::tet ΔrapZ λattB::*[*aadA, glmS'-lacZ*], *$strp^R$* , *F'*(*$lacI^q$*) | T4GT7 (IBPC750) → Z28, this work |

**Appendix Table S2**. Plasmids used in this study

| Name | Relevant structure[a] | Reference |
|---|---|---|
| pBAD33 | $P_{Ara}$, MCS 2, *cat*, ori p15A | (Guzman *et al.* 1995) |
| pBGG61 | *rapZ* under $P_{Ara}$ control in pBAD33 | (Göpel *et al.* 2013) |
| pBGG164 | *strep-rapZ* under $P_{tac}$ control, *lacI^q^*, *bla*, *ori* ColEI | (Lüttmann *et al.* 2012) |
| pBGG201 | fusion of *glmY*' (-238 to +22) to *lacZ* on plasmid pKES15 | (Reichenbach *et al.* 2009) |
| pBGG219 | *qseF-His*$_{10}$ under $P_{tac}$ control, *lacI*$^q$, *bla*, *ori* ColEI | (Reichenbach *et al.* 2009) |
| pBGG220 | *qseE'* (aa 196-475)-*His*$_{10}$ under $P_{tac}$ control, *lacI*$^q$, *bla*, *ori* ColEI | this work |
| pBGG237 | *strep-tag* under $P_{tac}$ control, *lacI*$^q$, *bla*, *ori* ColEI | (Lüttmann *et al.* 2012) |
| pBGG324 | fusion of *zraP*' (-289 to +41) to *lacZ* on plasmid pKES15 | (Reichenbach *et al.* 2009) |
| pBGG348 | encodes T25-RapZ in pKT25 | (Göpel *et al.* 2013) |
| pBGG349 | encodes T18-RapZ in pUT18C | (Göpel *et al.* 2013) |
| pBGG352 | encodes T25-QseF in pKT25 | this work |
| pBGG353 | encodes T18-QseF in pUT18C | this work |
| pBGG432 | encodes T25-RapZ in pKT25; *rapZ* with D182A exchange | (Gonzalez *et al.* 2017) |
| pBR-plac | IPTG inducible $P_{LlacO-1}$ promoter in pBR322, *bla*, starts sRNA transcription at +1 position | (Guillier & Gottesman 2006) |
| pDL43 | fusion of *glnA*' (-374 to +51) to *lacZ* on plasmid pKES15 | this work |
| pFDX4291 | operator-less $P_{tac}$, *sacB*-RBS, *cat*, *ori* pSC101 | (Kalamorz *et al.* 2007) |
| pFDX4324 | *rapZ* under $P_{tac}$ control in pFDX4291 | (Kalamorz *et al.* 2007) |
| pKES170 | *lacI*$^q$, $P_{tac}$, RBS *T7gene10*, MCS, *bla*, *ori* ColEI | (Lüttmann *et al.* 2009) |
| pKESK23 | *lacI*$^q$, $P_{tac}$::MCS, *neo*, *attP*, *aadA*, *ori* p15A | (Lüttmann *et al.* 2012) |
| pKT25 | $P_{lac}$::*cyaA* [*1-224*] (T25), MCS, *neo*, *ori* p15A | (Karimova *et al.* 1998) |
| pKT25-zip | encodes T25-GCN4 leucine zipper fusion in pKT25 | (Karimova *et al.* 1998) |
| pMK13 | *strep-kdpE* under $P_{tac}$ control in pKES170 | this work |
| pMM9 | *lacI*$^q$, $P_{tac}$:: *strep-kdpE*, *neo*, *ori p15A* | (Mörk-Mörkenstein *et al.* 2017) |
| pSD11 | encodes T18-RapZ (aa 1-152) fusion in pUT18C | (Gonzalez *et al.* 2017) |
| pSD12 | encodes T18-RapZ (aa 153-284) fusion in pUT18C | (Gonzalez *et al.* 2017) |
| pSD24 | *strep-rapZ-CTD* (aa 153-284) under $P_{tac}$ control, *lacI*$^q$, *bla*, *ori* ColEI | (Gonzalez *et al.* 2017) |
| pSD25 | *strep-rapZ-NTD* (aa 1-152) under $P_{tac}$ control, *lacI*$^q$, *bla*, *ori* ColEI | (Gonzalez *et al.* 2017) |
| pSD26 | *rapZ*$_{NTD}$ under $P_{Ara}$ control in pBAD33 | this work |
| pSD27 | *rapZ*$_{CTD}$ under $P_{Ara}$ control in pBAD33 | this work |
| pSD37 | encodes T18-RapZ in pUT18C; *rapZ* with D182A exchange | this work |
| pSD69 | *gcvB* in pBR-plac | this work |
| pUT18C | $P_{lac}$::*cyaA* [*225-399*] (T18), MCS, *bla*, *ori* ColEI | (Karimova *et al.* 1998) |
| pUT18C-zip | encodes T18-GCN4 leucine zipper fusion in pUT18C | (Karimova *et al.* 1998) |
| pYG30 | as pBGG61, but *rapZ* with K270A, K281A, R282A, K283A mutations | (Göpel *et al.* 2013) |
| pYG82 | *rapZ*$_{quad}$ under $P_{tac}$ control in pFDX4291 | this work |
| pYG83 | *glmY* in pBR-plac | (Göpel *et al.* 2016) |
| pYG84 | *glmZ* in pBR-plac | (Göpel *et al.* 2016) |
| pYG89 | *qseF* with cognate *RBS* under $P_{tac}$ control in pKESK23 | (Göpel & Görke 2018) |
| pYG90 | as pYG89, but *qseF* with D56E exchange | (Göpel & Görke 2018) |
| pYG93 | as pYG89, but *qseF* with D56A exchange | (Göpel & Görke 2018) |
| pYG199 | encodes T25-QseE in pKT25 | (Göpel & Görke 2018) |
| pYG246 | encodes T18-QseE in pUT18C | (Göpel & Görke 2018) |

[a]*ori*: origin of replication; *RBS*: ribosomal binding site, MCS: multiple cloning site

**Appendix Table S3**. Oligonucleotides used in this study.

| Primer | Sequence[a] | Res. Sites | Position[b] |
|---|---|---|---|
| BG230 | GTAGATGCTCATTCCATCTC | | *glmZ* 1 to 20 |
| BG231 | CTAATACGACTCACTATAGGGAGAAAACAGGTCTGTATGACAAC | | *glmZ* 172 to 152 |
| BG260 | AGTGGCTCATTCACCGAC | | *glmY* 1 to 18 |
| BG261 | CTAATACGACTCACTATAGGGAGATAAGGCGGTGCCTAACTC | | *glmY* 150 to 131 |
| BG287 | TGCCTGGCGGCCGTAG | | *rrfD* 1 to 16 |
| BG288 | CTAATACGACTCACTATAGGGAGAGCCTGGCAGTTCCCTAC | | *rrfD* 118 to 102 |
| BG397 | TGGCTGCAGTCTAGATTATCATGGTTTACGTTTTTCCAGCG | PstI, XbaI | *rapZ* 855 to 833 |
| BG444 | CTAATACGACTCACTATAGGGAGAGTAGATGCTCATTCCATCTCTTATG | | *glmZ* 1 to 25 |
| BG445 | AAAAAAACGCCTGCTCTTATTACGGAGC | | *glmZ* 207 to 180 |
| BG446 | CTAATACGACTCACTATAGGGAGAAGTGGCTCATTCACCGACTTATGTC | | *glmY* 1 to 25 |
| BG448 | AAGGCGGTGCCTAACTCGACG | | *glmY* 148 to 128 |
| BG486 | CTCGTACTCATATGATTATCGGGCCGGTGAAAAATATC | NdeI | *qseE* 586 to 612 |
| BG487 | GGCTCTAGATTTCGTGTTTTTCGACGACGG | XbaI | *qseE* 1425 to 1405 |
| BG640 | GCGTCTAGAGATGAGCCATAAACCTGCGC | XbaI | *qseF* 1 to 19 |
| BG642 | CGCGGTACCTCATTCCTTGAAATCGTTTGC | KpnI | *qseF* 1335 to 1315 |
| BG934 | GCACGCGTCGACAACTTTGCCTCAGGCATTAG | SalI | *glnA* -374 to -353 |
| BG935 | GGCTCTAGAAACTTCACTTCGTGCTCG | XbaI | *glnA* 51 to 33 |
| BG1049 | GGCGAGCTCGTGAGGAGAAACAGTACATGGTACTGATGATCGTCAGCG | SacI | *rapZ* -17 to +22 |
| BG1301 | ATCTGCTAAACGTAACACATAACGCCAATTCATTCCTTGAAATCGTTTGC CATATGAATATCCTCCTTAGTTCCTATTCC | | *qseF* 1364 to 1315 |
| BG1358 | GCGTCTAGATTACAGACGGGTACGCAGCATTT | XbaI | *rapZ* 456 to 437 |
| BG1359 | GGCGAGCTCGTGAGGAGAAACAGTACATGCTGGGTAAACGTGAACGCG AAC | SacI | *rapZ* -17 to -1 and +457 to +478 |
| BG1380 | CGATGACGTCACTTCCTGAGCCGGAACGAAAAG | AatII | *gcvB* 1 to 23 |
| BG1381 | CGTACGAATTCGTAATTCGCGATCGCAAGGTAA | EcoRI | *gcvB* 226 to 205 |
| BG1390 | TTGAAACGCTGGCCCGTTTTTCCCCGCTCATTACGACAACTGGTATGTA GGCTGGAGCTGCTTCG | | *qseE* 1 to 45 |
| BG1792 | CTAATACGACTCACTATAGGGAGAACTTCCTGAGCCGGAACGAAAAG | | *gcvB* 1 to 23 |
| BG1793 | AAAAAAAGCACCGCAATTAGGCGG | | *gcvB* 205 to 182 |

[a]Restriction sites are underlined. The recognition site for T7 RNA polymerase is underlined by a dashed line. [b]Positions are relative to the first nucleotide of the respective gene as annotated in the EcoCyc database (Keseler *et al.* 2017).

# Appendix Supplementary Materials and Methods

## Strains, plasmids and oligonucleotides.

Strains and plasmids are listed in Appendix Tables S1 and S2, respectively. Oligonucleotides are documented in Appendix Table S3. Established alleles were moved between strains by general transduction using phage T4GT7 (Wilson *et al.* 1979). Resistance markers flanked by FLP recombinase sites were subsequently removed from the chromosome by using the temperature sensitive plasmid pCP20 (Datsenko & Wanner 2000). When not moved by transduction, *lacZ* reporter fusions were first established on plasmids and subsequently integrated into the *λattB* site on the chromosome using a site-specific recombination system (Diederich *et al.* 1992) as described before (Göpel *et al.* 2011). The deletion of the *qseEGF* operon in strain Z967 was constructed using the λ red recombination system, oligonucleotides BG1390/BG1301 and plasmid pKD3 as described (Datsenko & Wanner 2000). The genotypes of all strains were confirmed by analytic PCR.

## Construction of plasmids.

Plasmid pYG82 expressing $rapZ_{quad}$ from an operator-less $P_{tac}$ promoter was constructed by substitution of the BamHI-PstI fragment of plasmid pFDX4224 comprising the 3' end of *rapZ* with the BamHI-XbaI fragment of plasmid pYG29 (Göpel *et al.* 2013). For construction of pBAD33 derivatives directing expression of *rapZ-CTD* and *rapZ-NTD* from the $P_{Ara}$ promoter, respectively, the *rapZ* variants were amplified using primers BG1049/BG1358 ($rapZ_{NTD}$) and BG1359/BG397 ($rapZ_{CTD}$) and PCR fragments were inserted between the SacI/XbaI sites on pBAD33 resulting in plasmids pSD26 and pSD27. Plasmid pDL43 carrying a transcriptional reporter fusion of *lacZ* to the *glnA'* 5' region (-374 to +51), was constructed by amplification of the *glnA'* fragment using oligonucleotides BG934/BG935 and subsequent insertion of the resulting PCR fragment between the SalI/XbaI sites on plasmid pKES15. Plasmid pSD69 carries sRNA *gcvB* under $P_{LlacO\text{-}1}$ promoter control. For its construction, *gcvB* was amplified using oligonucleotides BG1380/BG1381 and subsequently cloned between the AatII/EcoRI sites on plasmid pBR-plac. Plasmid pMK13 allows for overexpression of *strep-kdpE*. It was constructed by ligating the ApaI-PstI fragment of plasmid pMM9 comprising *strep-kdpE* with vector pKES170 that was digested with the same enzymes. Plasmid pBGG220 triggers overproduction of the C-terminally $His_{10}$-

tagged cytoplasmic part (aa 196-475) of kinase QseE. For its construction the corresponding sequence of *qseE* was amplified using oligonucleotides BG486/BG487 and the resulting PCR fragment was cloned between the NdeI/XbaI sites on plasmid pKES170. For construction of plasmids pBGG352 and pBGG353, *qseF* was amplified using oligonucleotides BG640/BG642 and subsequently inserted between the XbaI and KpnI sites on plasmids pKT25 and pUT18C, respectively. Plasmid pSD37 encoding the T18-RapZ (Asp182Ala) fusion protein was constructed by moving the XbaI-KpnI fragment of plasmid pBGG432 to pUT18C.

### Protein purification.

Strep- and $His_{10}$-tagged proteins were purified by StrepTactin and Ni-NTA affinity chromatography, respectively. The following plasmids were used for protein overproduction: pBGG164 (Strep-RapZ), pSD24 (Strep-RapZ-CTD), pSD25 (Strep-RapZ-NTD), pMK13 (Strep-KdpE), pBGG219 (QseF-$His_{10}$) and pBGG220 (QseE'-$His_{10}$). For purification of proteins from GlcN6P replete or depleted cells, the *ΔglmS* mutant Z904 carrying the required overproduction plasmid was used. Cells were grown at 37°C in LB supplemented either with 0.2 % GlcN or an equivalent volume of $H_2O$. Following 1 h growth, 1 mM IPTG was added for induction of protein synthesis, and growth was continued until the GlcN6P replete cultures reached $OD_{600}$ ~ 2.0. Cells were washed and resuspended in buffer W (150 mM NaCl, 100 mM Tris-HCl, 1 mM EDTA, pH 8.0) for purification of Strep-tagged proteins and in ZAP buffer (50 mM Tris-HCl, 200 mM NaCl, pH 7.5) for purification of $His_{10}$-tagged proteins. Cells were subsequently lysed using a sonicator (pulses of 10 s with 80 % power (MS-2/70)) or a French pressure cell (15.000 psi). Following centrifugation steps (4000 rpm, 4°C, 20 min, Eppendorf, 5810R and 35.000 rpm, 4°C, 1 h, Beckman L-70), cleared lysates were loaded on poly-prep chromatography columns (Biorad) containing either 1 ml pre-equilibrated StrepTactin sepharose matrix or 3 ml Ni-NTA matrix (IBA, Göttingen) suspension per 1 l culture. The StrepTactin matrix was washed 4× with 10 ml buffer W and the proteins of interest were finally eluted in three steps using 3 × 1 ml buffer E (150 mM NaCl, 100 mM Tris- HCl, 1 mM EDTA, 2.5 mM D-desthiobiotin (IBA), pH 8.0). $His_{10}$-tagged proteins were eluted using incremental concentrations of imidazole (5 mM, 10 mM, 25 mM, 50 mM, 80mM, 125 mM, 250 mM, 500 mM imidazole prepared in ZAP buffer). Protein concentrations were determined by Bradford assay and confirmed by SDS-PAGE. Following dialysis (2 × 16 h, 4°C) in dialysis buffer (10 mM Tris-HCl, pH 7.0, 100 mM KCl, 10 mM $MgCl_2$, 2 mM β-mercaptoethanol, 10 % glycerol), proteins were aliquoted, frozen in liquid nitrogen and stored at -80°C until use.

## *In vitro* transcription and radioactive labelling of sRNA.

Radiolabeled and non-radiolabeled sRNAs were obtained by *in vitro* transcription of appropriate PCR fragments using T7 RNA polymerase. The PCR templates for *in vitro* transcription were generated using oligonucleotides and BG446/BG448 (GlmY*), BG444/BG445 (full-length GlmZ) and BG1792/BG1793 (GcvB). For sRNA labeling, 1 μg of the respective PCR fragment was used in a 20 μl *in vitro* transcription reaction containing 50 units T7 RNA polymerase (NEB), 40 units RiboLock RNase inhibitor (Thermo Fisher Scientific), ATP, CTP, GTP (0.5 mM each), 0.005 mM UTP and 20 μCi $\alpha$-$^{32}$P-UTP (6000 μCi/mMol; Hartmann Analytic) in 1×RNA Pol reaction buffer. The reaction was incubated at 37ºC for at least 5 h followed by removal of unincorporated nucleotides using Illustra Microspin G-50 columns (GE Healthcare). After addition of 2× RNA loading dye (95% formamide, 0.5 mM EDTA, 0.025% SDS, 0.025% bromophenol blue, 0.025% xylene cyanol), RNA was denatured (95°C, 1 min), chilled and subsequently electrophoretically separated on a denaturing gel (7M urea, 6% acrylamide, 1× TBE) using 1× TBE as running buffer. Subsequently, the wet gel was subjected to phospho-imaging, thus enabling excision of the correct RNA. RNA was extracted from the gel piece by incubation in 400 μl RNA elution buffer (20 mM Tris-HCl pH 7.5, 0.25 M sodium acetate, 1 mM EDTA, 0.25% w/v SDS), shock freezing on dry ice followed by overnight incubation at RT (Nilsen 2013). RNA was further extracted via phenol:$CHCl_3$:isoamyl alcohol (25:24:1) and precipitated with ethanol:3M sodium acetate pH 5.2 (30:1) at -20°C for at least 2 h and dissolved in 20 µl $H_2O$. Unlabeled sRNAs were produced using the same protocol, but with equal NTP concentrations (5 mM each NTP). Furthermore the nucleotide removal step was omitted and for gel purification the denaturing gel was stained with ethidium bromide prior to excision of the sRNAs.

## Appendix Supplementary References

Abel S, Chien P, Wassmann P, Schirmer T, Kaever V, Laub MT, Baker TA, Jenal U. 2011. Regulatory cohesion of cell cycle and cell differentiation through interlinked phosphorylation and second messenger networks. *Mol Cell* **43**: 550-560.

Baba T, Ara T, Hasegawa M, Takai Y, Okumura Y, Baba M, Datsenko KA, Tomita M, Wanner BL, Mori H. 2006. Construction of *Escherichia coli* K-12 in-frame, single-gene knockout mutants: the Keio collection. *Mol Syst Biol* **2**: 1-11.

Datsenko KA, Wanner BL. 2000. One-step inactivation of chromosomal genes in *Escherichia coli* K-12 using PCR products. *Proc Natl Acad Sci U S A* **97**: 6640-6645.

Diederich L, Rasmussen LJ, Messer W. 1992. New cloning vectors for integration in the *lambda* attachment site *attB* of the *Escherichia coli* chromosome. *Plasmid* **28**: 14-24.

Gonzalez GM, Durica-Mitic S, Hardwick SW, Moncrieffe MC, Resch M, Neumann P, Ficner R, Görke B, Luisi BF. 2017. Structural insights into RapZ-mediated regulation of bacterial amino-sugar metabolism. *Nucleic Acids Res* **45**: 10845-10860.

Göpel Y, Lüttmann D, Heroven AK, Reichenbach B, Dersch P, Görke B. 2011. Common and divergent features in transcriptional control of the homologous small RNAs GlmY and GlmZ in *Enterobacteriaceae*. *Nucleic Acids Res* **39**: 1294-1309.

Göpel Y, Papenfort K, Reichenbach B, Vogel J, Görke B. 2013. Targeted decay of a regulatory small RNA by an adaptor protein for RNase E and counteraction by an anti-adaptor RNA. *Genes Dev* **27**: 552-564.

Göpel Y, Khan MA, Görke B. 2016. Domain swapping between homologous bacterial small RNAs dissects processing and Hfq binding determinants and uncovers an aptamer for conditional RNase E cleavage. *Nucleic Acids Res* **44**: 824-837.

Göpel Y, Görke B. 2018. Interaction of lipoprotein QseG with sensor kinase QseE in the periplasm controls the phosphorylation state of the two-component system QseE/QseF in *Escherichia coli*. *PLoS Genet* **14**: e1007547.

Guillier M, Gottesman S. 2006. Remodelling of the *Escherichia coli* outer membrane by two small regulatory RNAs. *Mol Microbiol* **59**: 231-247.

Guzman LM, Belin D, Carson MJ, Beckwith J. 1995. Tight regulation, modulation, and high-level expression by vectors containing the arabinose $P_{BAD}$ promoter. *J Bacteriol* **177**: 4121-4130.

Kalamorz F, Reichenbach B, März W, Rak B, Görke B. 2007. Feedback control of glucosamine-6-phosphate synthase GlmS expression depends on the small RNA GlmZ and involves the novel protein YhbJ in *Escherichia coli*. *Mol Microbiol* **65**: 1518-1533.

Karimova G, Pidoux J, Ullmann A, Ladant D. 1998. A bacterial two-hybrid system based on a reconstituted signal transduction pathway. *Proc Natl Acad Sci U S A* **95**: 5752-5756.

Keseler IM, Mackie A, Santos-Zavaleta A, Billington R, Bonavides-Martinez C, Caspi R, Fulcher C, Gama-Castro S, Kothari A, Krummenacker M, Latendresse M, Muniz-Rascado L, Ong Q, Paley S, Peralta-Gil M, Subhraveti P, Velazquez-Ramirez DA, Weaver D, Collado-

Vides J, Paulsen I *et al.* 2017. The EcoCyc database: reflecting new knowledge about *Escherichia coli* K-12. *Nucleic Acids Res* **45**: D543-D550.

Khan MA, Göpel Y, Milewski S, Görke B. 2016. Two Small RNAs Conserved in *Enterobacteriaceae* Provide Intrinsic Resistance to Antibiotics Targeting the Cell Wall Biosynthesis Enzyme Glucosamine-6-Phosphate Synthase. *Front Microbiol* **7**: 908.

Lüttmann D, Heermann R, Zimmer B, Hillmann A, Rampp IS, Jung K, Görke B. 2009. Stimulation of the potassium sensor KdpD kinase activity by interaction with the phosphotransferase protein $IIA^{Ntr}$ in *Escherichia coli*. *Mol Microbiol* **72**: 978-994.

Lüttmann D, Göpel Y, Görke B. 2012. The phosphotransferase protein $EIIA^{Ntr}$ modulates the phosphate starvation response through interaction with histidine kinase PhoR in *Escherichia coli*. *Mol Microbiol* **86**: 96-110.

Magasanik B. 1993. The regulation of nitrogen utilization in enteric bacteria. *J Cell Biochem* **51**: 34-40.

Mörk-Mörkenstein M, Heermann R, Göpel Y, Jung K, Görke B. 2017. Non-canonical activation of histidine kinase KdpD by phosphotransferase protein PtsN through interaction with the transmitter domain. *Mol Microbiol* **106**: 54-73.

Neidhardt FC, Bloch PL, Smith DF. 1974. Culture medium for enterobacteria. *J Bacteriol* **119**: 736-747.

Nilsen TW. 2013. Gel purification of RNA. *Cold Spring Harb Protoc* **2013**: 180-183.

Plumbridge J, Vimr E. 1999. Convergent pathways for utilization of the amino sugars N-acetylglucosamine, N-acetylmannosamine, and N-acetylneuraminic acid by *Escherichia coli*. *J Bacteriol* **181**: 47-54.

Reichenbach B, Göpel Y, Görke B. 2009. Dual control by perfectly overlapping sigma 54- and sigma 70- promoters adjusts small RNA GlmY expression to different environmental signals. *Mol Microbiol* **74**: 1054-1070.

Schnetz K, Stülke J, Gertz S, Krüger S, Krieg M, Hecker M, Rak B. 1996. LicT, a *Bacillus subtilis* transcriptional antiterminator protein of the BglG family. *J Bacteriol* **178**: 1971-1979.

Venkatesh GR, Kembou Koungni FC, Paukner A, Stratmann T, Blissenbach B, Schnetz K. 2010. BglJ-RcsB heterodimers relieve repression of the *Escherichia coli bgl* operon by H-NS. *J Bacteriol* **192**: 6456-6464.

Wilson GG, Young KY, Edlin GJ, Konigsberg W. 1979. High-frequency generalised transduction by bacteriophage T4. *Nature* **280**: 80-82.
