# Supplementary material for: Small RNA‐binding protein RapZ mediates cell envelope precursor sensing and signaling in Escherichia coli
Source: EMBO J. 2020 Feb 17;39(6):e103848. doi: 10.15252/embj.2019103848 (PMC7073468; doi:10.15252/embj.2019103848)
Supplement: Supplementary file 2 — Expanded View Figures PDF [file EMBJ-39-e103848-s002.pdf]

Expanded View Figures

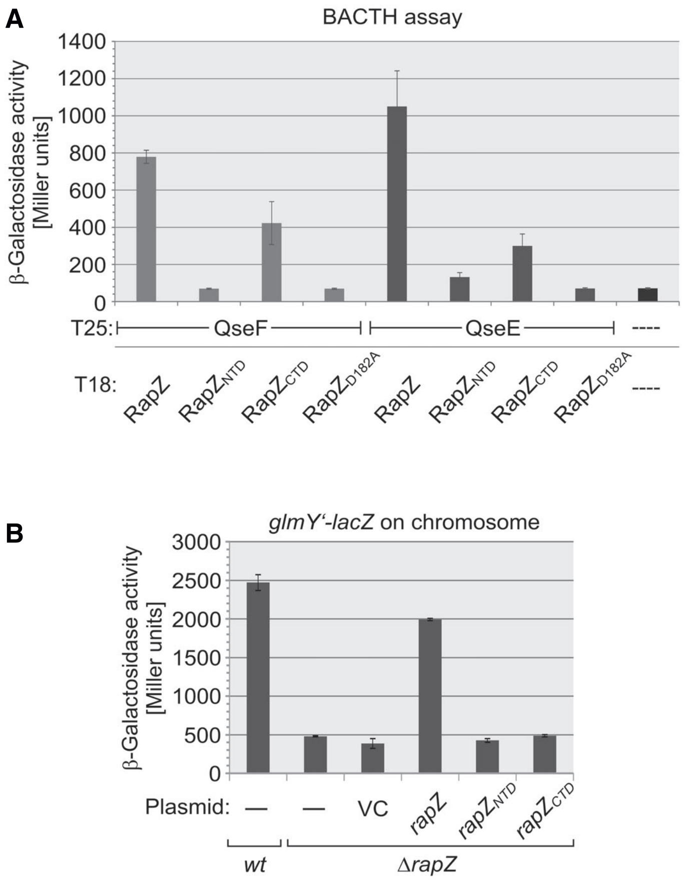

Figure EV1. The separated globular domains of RapZ are impaired in binding QseE and QseF and fail to activate *glmY* expression.

A BACTH assay addressing interaction of RapZ variants with QseF and QseE. The following plasmid combinations were tested in reporter strain RH785 (columns from left to right): pBGG352/pBGG349, pBGG352/pSD11, pBGG352/pSD12, pBGG352/pSD37, pYG199/pBGG349, pYG199/pSD11, pYG199/pSD12, pYG199/pSD37, and pKT25/pUT18C (negative control).

B Complementation experiment analyzing the requirement of the globular domains of RapZ for *glmY* expression. Strains Z197 and the *ΔrapZ* mutant Z225 were used. The following plasmids were tested in strain Z225: pBAD33 (vector control = VC), pBGG61 (*rapZ*), pSD26 (*rapZ-NTD*), and pSD27 (*rapZ-CTD*). 0.2% arabinose was added to the cultures to induce expression of the *rapZ* variants from the P_{Ara} promoter.

Data information: In (A) and (B), β-galactosidase activities are presented as mean ± SD, $n = 3$. Source data are available online for this figure.

© 2020 The Authors

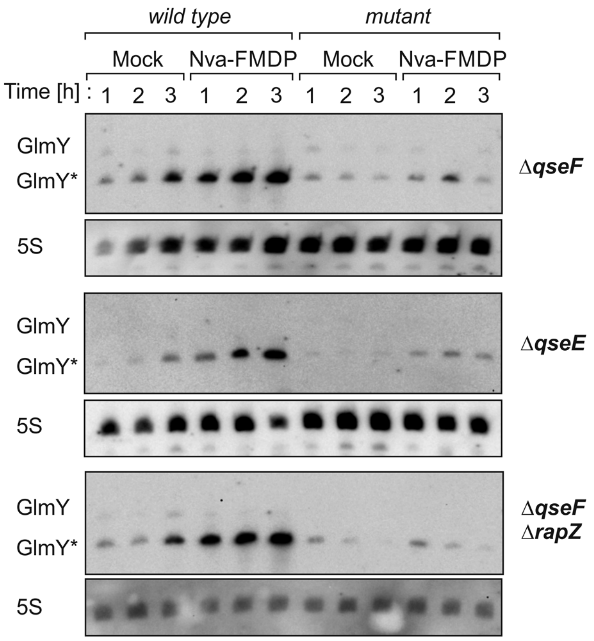

Figure EV2. Upon GlcN6P depletion, GlmY* still accumulates to a minor extent in *ΔqseF* and *ΔqseE* mutants and this effect requires *rapZ*.

Northern blots comparing GlmY levels in MG1655 derivative strains. The *wild-type* strain Z854, the *ΔqseF* mutant Z1081, the *ΔqseE* mutant Z1082, and the *ΔqseF ΔrapZ* double mutant Z1083 were assessed under normal growth ("mock") and GlcN6P starvation ("Nva-FMDP") conditions, respectively. Bacteria were grown and analyzed as described for Fig 1A. Total RNAs were subjected to Northern analysis using probes directed against GlmY and 5S rRNA. Total RNA isolated from the various mutants was analyzed on the right halves of the various blots alongside *wild-type* RNA samples (left halves of the blots) for direct comparison. Analysis of the isogenic *ΔrapZ* mutant under the same conditions is provided in Appendix Fig S2 for comparison.
Source data are available online for this figure.

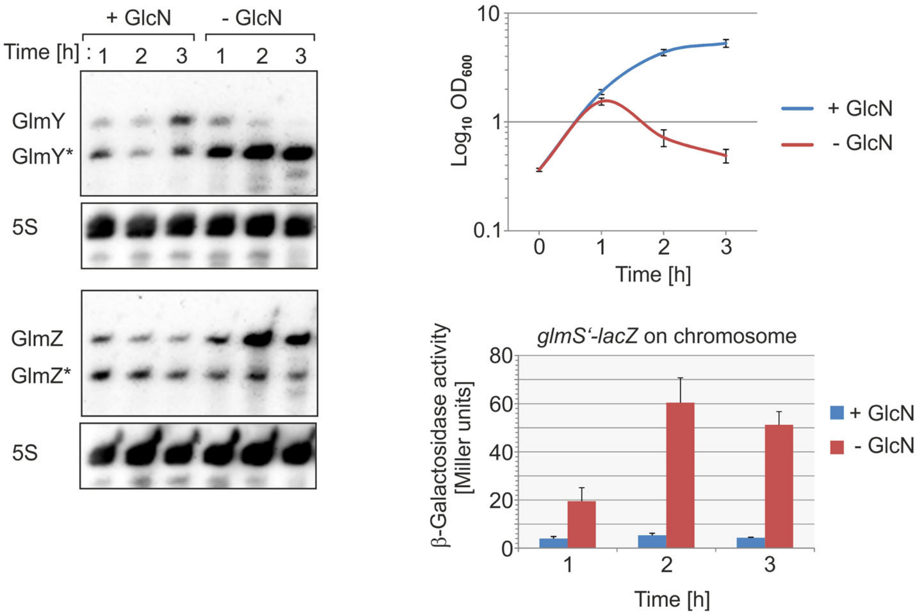

Figure EV3. Usage of a *ΔglmS* mutation to study the impact of GlcN6P on the regulatory GlmY/GlmZ/*glmS* circuit.

Strain Z904 (*ΔglmS*; MG1655 derivative) carrying a *glmS'-lacZ* reporter fusion on the chromosome was grown in LB supplemented with 0.2% GlcN until OD_{600} = 0.3. Subsequently, the culture was split, cells were washed, and growth was continued in the presence or absence of 0.2% GlcN. Samples were harvested at hourly intervals and used for determination of β-galactosidase activities (right, bottom) and extraction of total RNA, which was analyzed by Northern blotting using probes specific for GlmY, GlmZ, and 5S rRNA (left). The corresponding growth curves are provided (right, top). The time = 0 h refers to the time of splitting the culture.
Data information: β-galactosidase activities and OD_{600} values underlying the growth curves are presented as mean ± SD, n = 3.
Source data are available online for this figure.

 © 2020 The Authors

 © 2020 The Authors

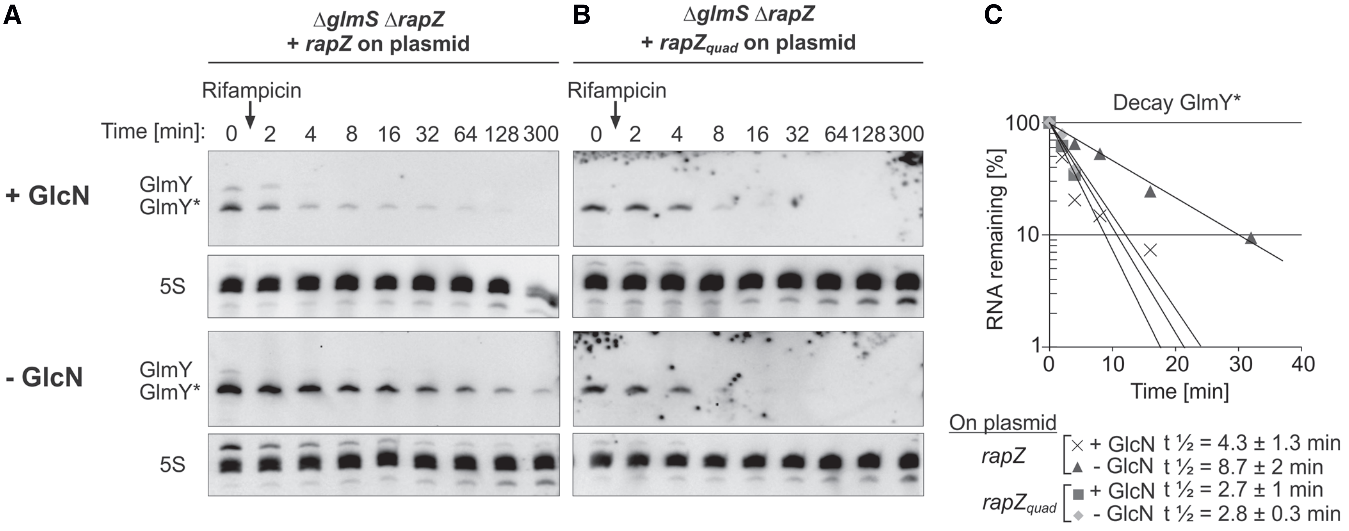

Figure EV4. Stabilization of GlmY* upon GlcN6P starvation requires the RNA-binding activity of RapZ.

Strain Z939 (*ΔglmS ΔrapZ*) was used, which harbored a plasmid transcribing either *rapZ* or $rapZ_{quad}$ from the P_{Ara} promoter. $RapZ_{quad}$ comprises alanine substitutions of four amino acid residues (Lys270, Lys281, Arg282, and Lys283) abrogating RNA-binding activity. The bacteria were grown and analyzed as described for Fig 6. For induction of *rapZ* expression, 0.2% arabinose was added to the pre-cultures used for inoculation of the test cultures containing or lacking 0.2% GlcN.

A Analysis of strain Z939 harboring plasmid pBGG61 transcribing *wild-type rapZ*.

B Analysis of strain Z939 harboring plasmid pYG30 transcribing $rapZ_{quad}$.

C Semi-logarithmic plots of GlmY* decay for half-life determination.

Data information: In (C), data are presented as mean, $n = 2$.

Source data are available online for this figure.
