# Supplementary figures and images for: Small RNA‐binding protein RapZ mediates cell envelope precursor sensing and signaling in Escherichia coli
Source: EMBO J. 2020 Feb 17;39(6):e103848. doi: 10.15252/embj.2019103848 (PMC7073468; doi:10.15252/embj.2019103848)

# Source data\_Northern blots\_Khan\_Fig1A

Final Figure was prepared by clipping the boxed areas

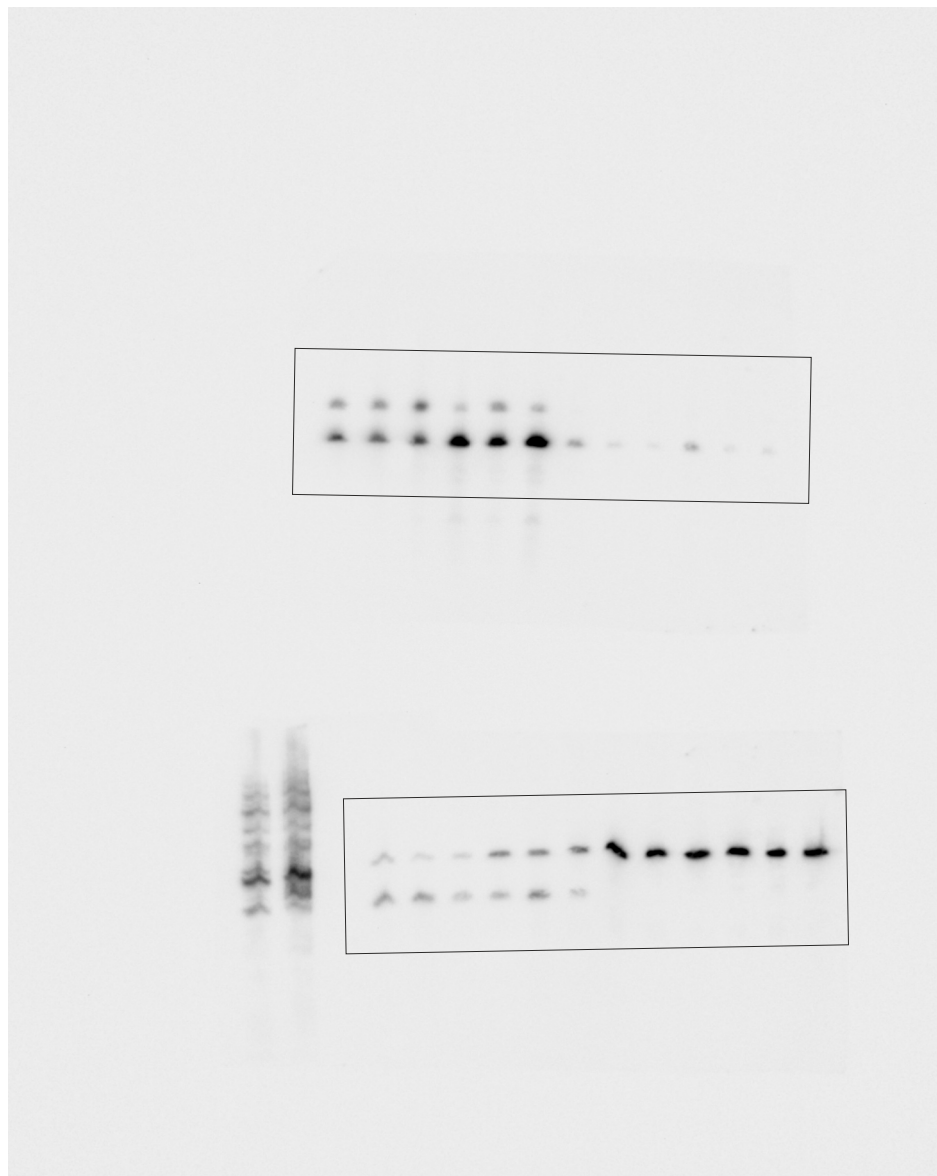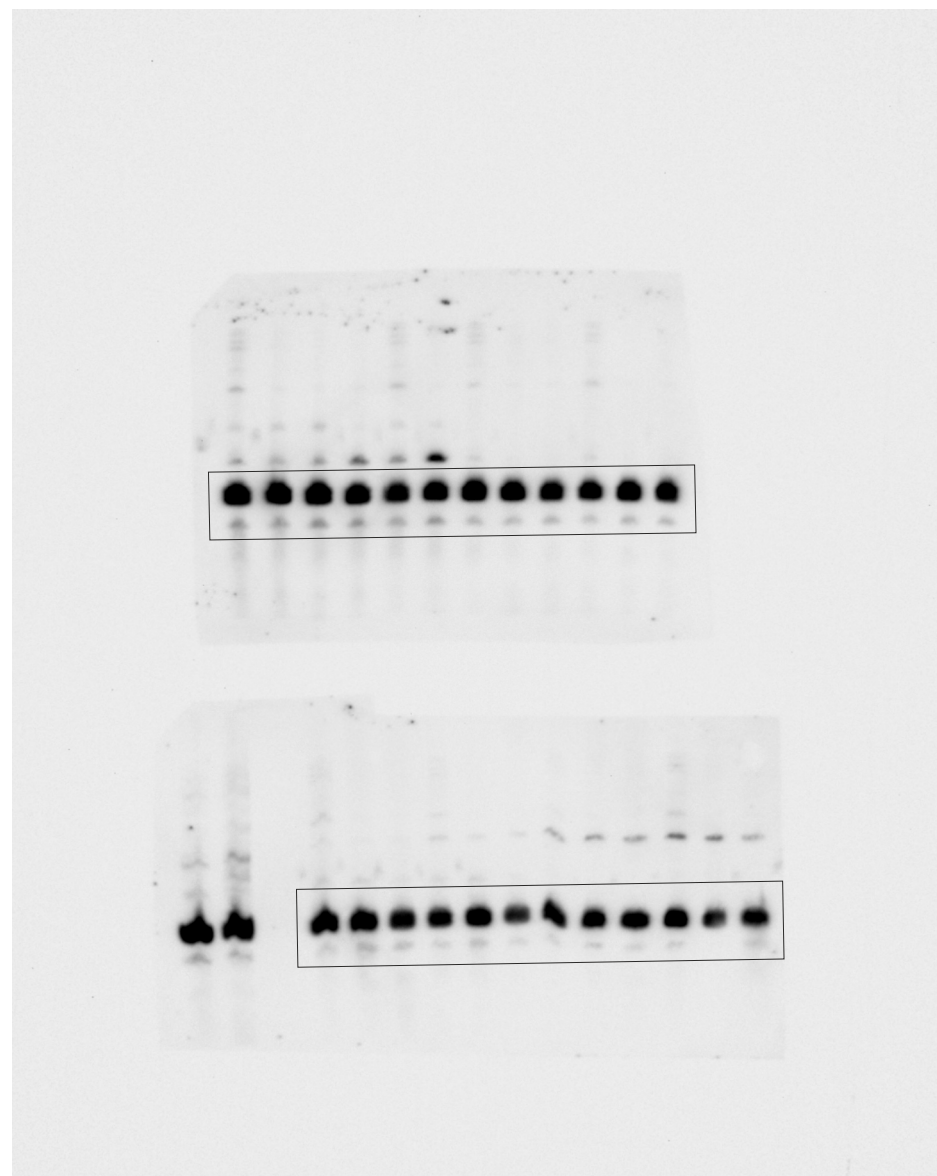

Supplement: Supplementary file 5 — Source Data for Figure 1 [file EMBJ-39-e103848-s003.zip › embj2019103848-SourceDataForFigure1/Source_data_Fig_1A_Northern_blots.pdf]

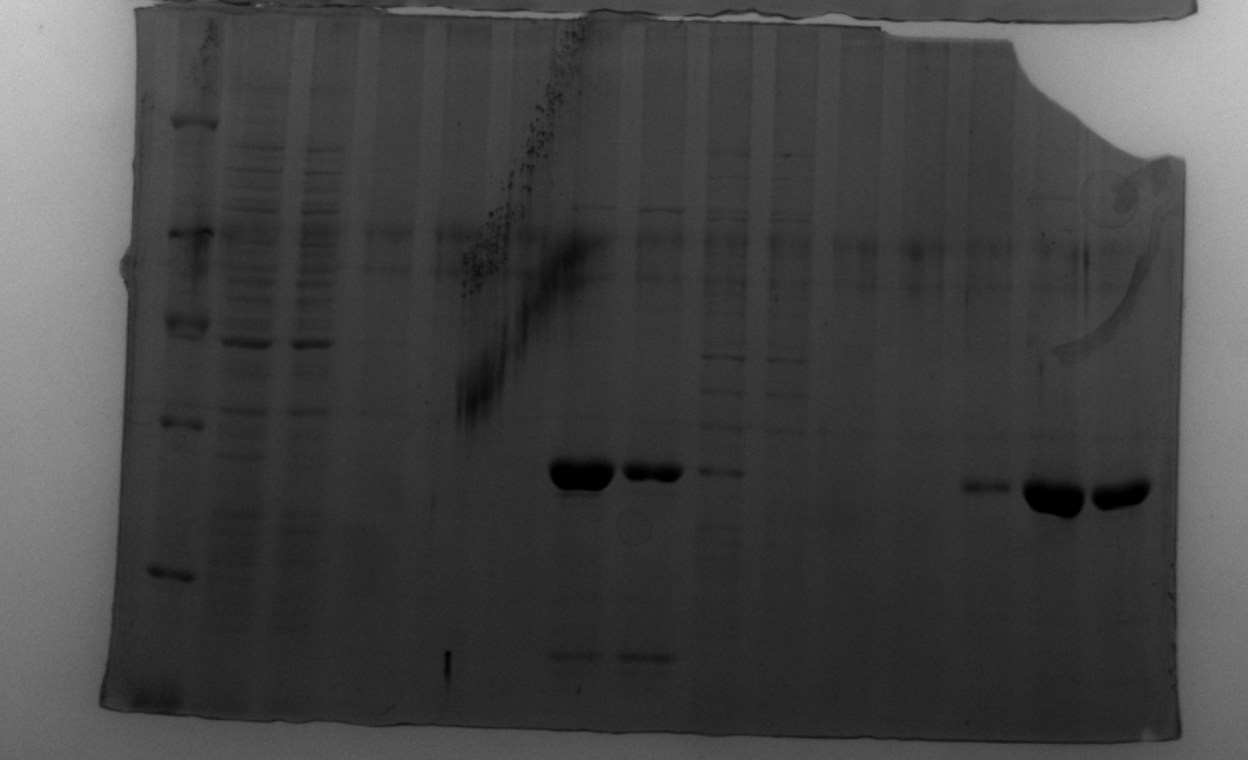

Supplement: Supplementary file 5 — Source Data for Figure 1 [file EMBJ-39-e103848-s003.zip › embj2019103848-SourceDataForFigure1/Source_data_Fig_1B.tif]

Fig3D

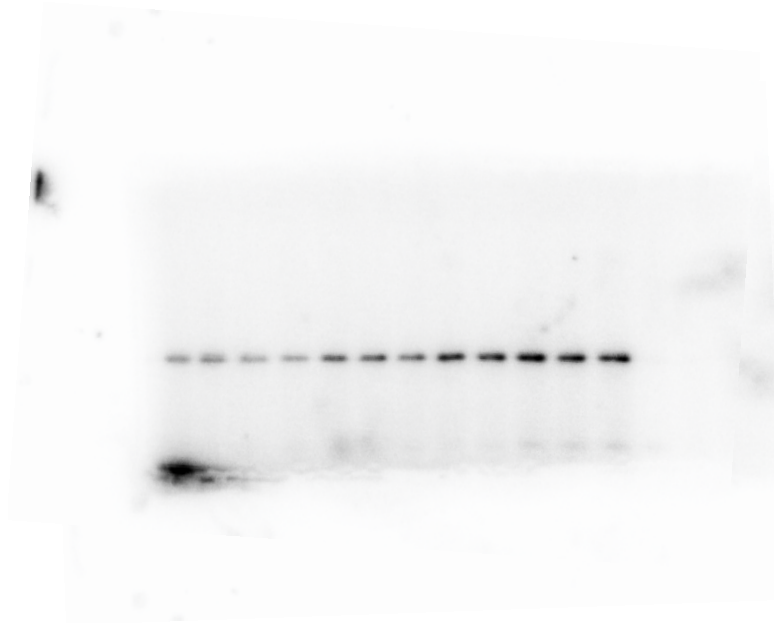

Fig3E

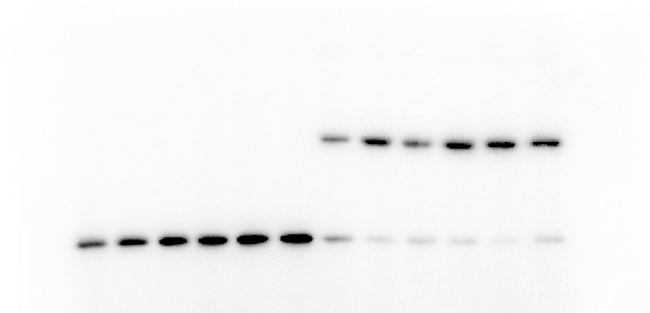

Fig3F

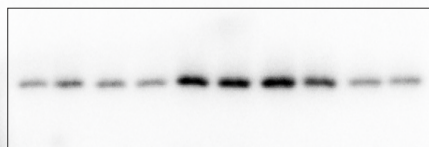

Supplement: Supplementary file 7 — Source Data for Figure 3 [file EMBJ-39-e103848-s005.zip › Source_data_Fig_3D-3F_phosphorylation_assays.pdf]

## Source data\_Khan\_Fig4C

Fig4C, lanes 1-11

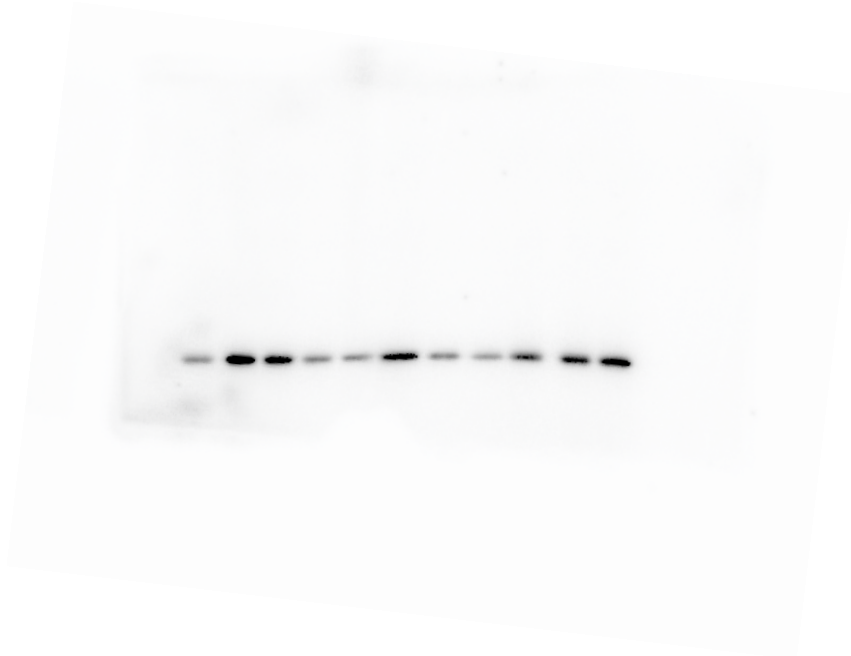

Fig. 4C, lanes 12-16

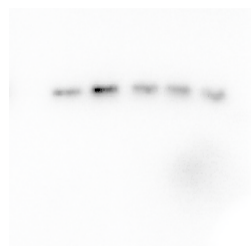

Supplement: Supplementary file 8 — Source Data for Figure 4 [file EMBJ-39-e103848-s006.zip › Source_data_Fig_4C_phosphorylation_assays.pdf]

# Source data\_Khan\_Fig6D

Fig6D, left panel

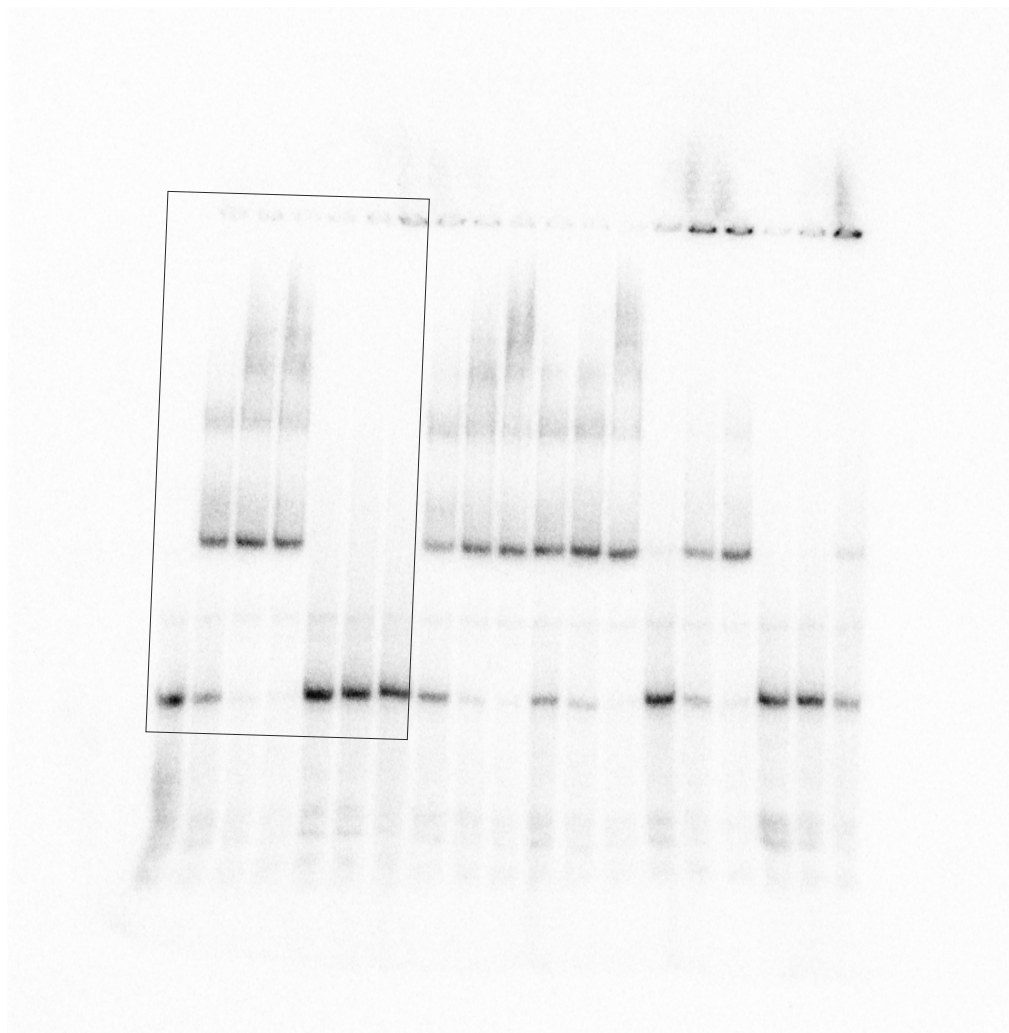

Fig6D, right panel

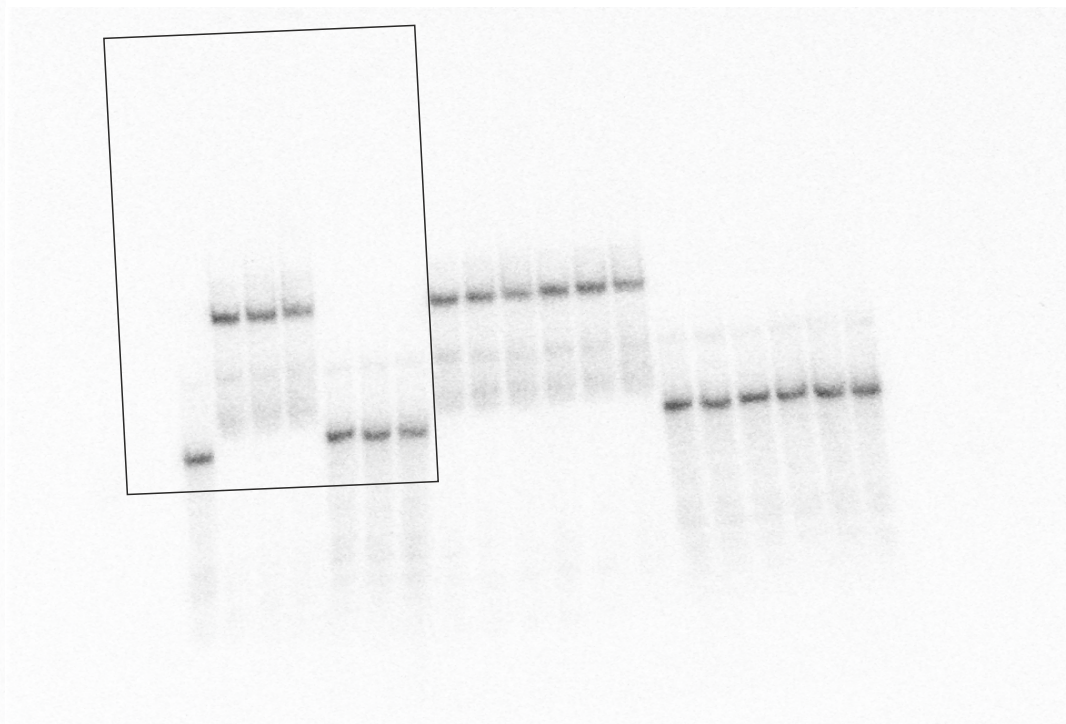

Supplement: Supplementary file 10 — Source Data for Figure 6 [file EMBJ-39-e103848-s008.zip › Source_data_Fig_6D_EMSAs.pdf]

Source data\_Khan\_Fig6E

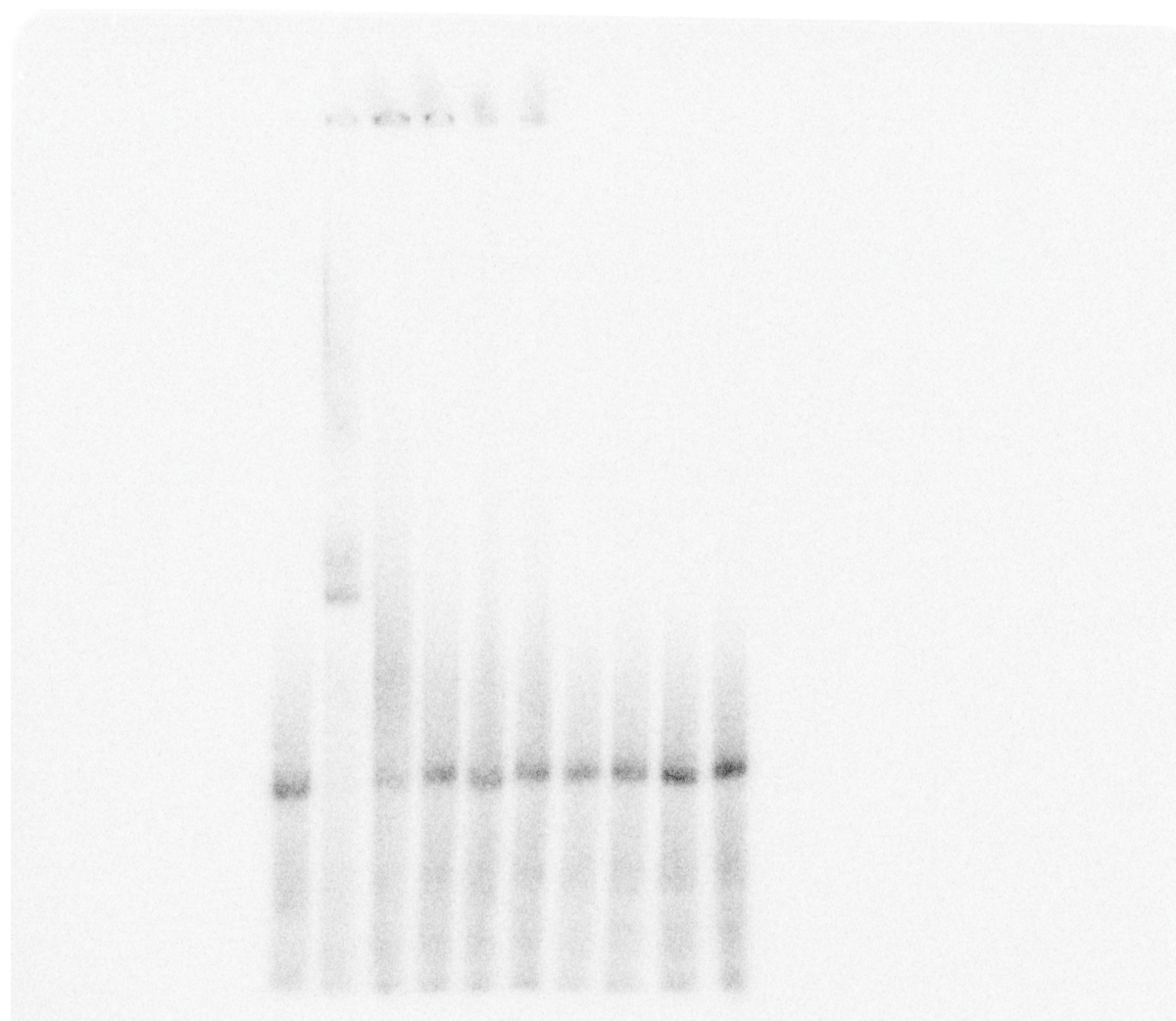

Supplement: Supplementary file 10 — Source Data for Figure 6 [file EMBJ-39-e103848-s008.zip › Source_data_Fig_6E_EMSAs.pdf]

Fig6F, left panel

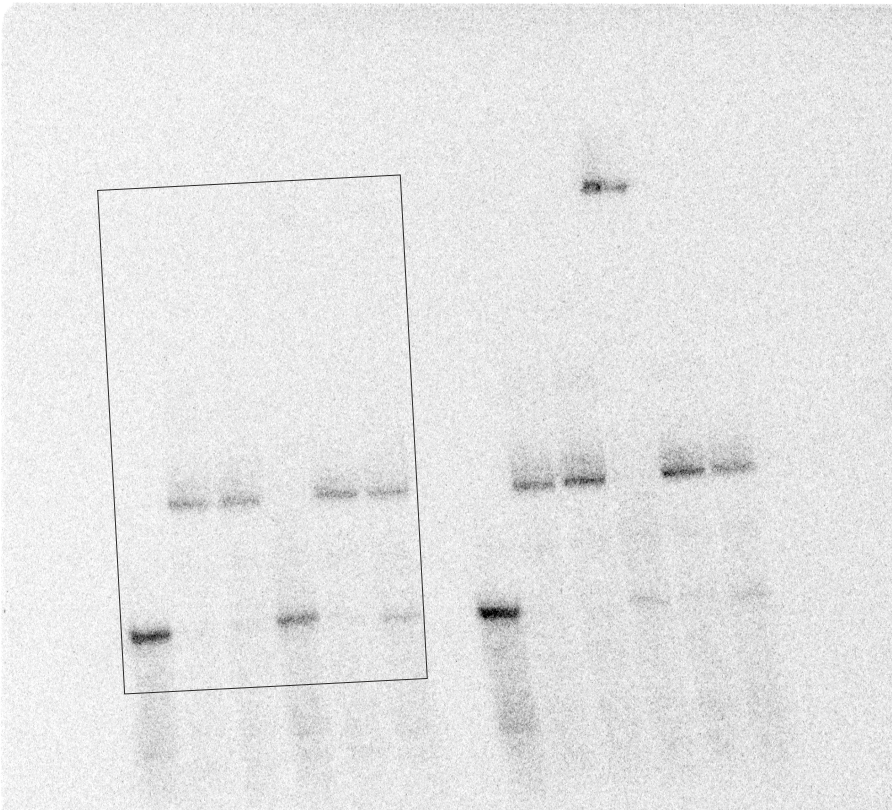

Fig6F, right panel

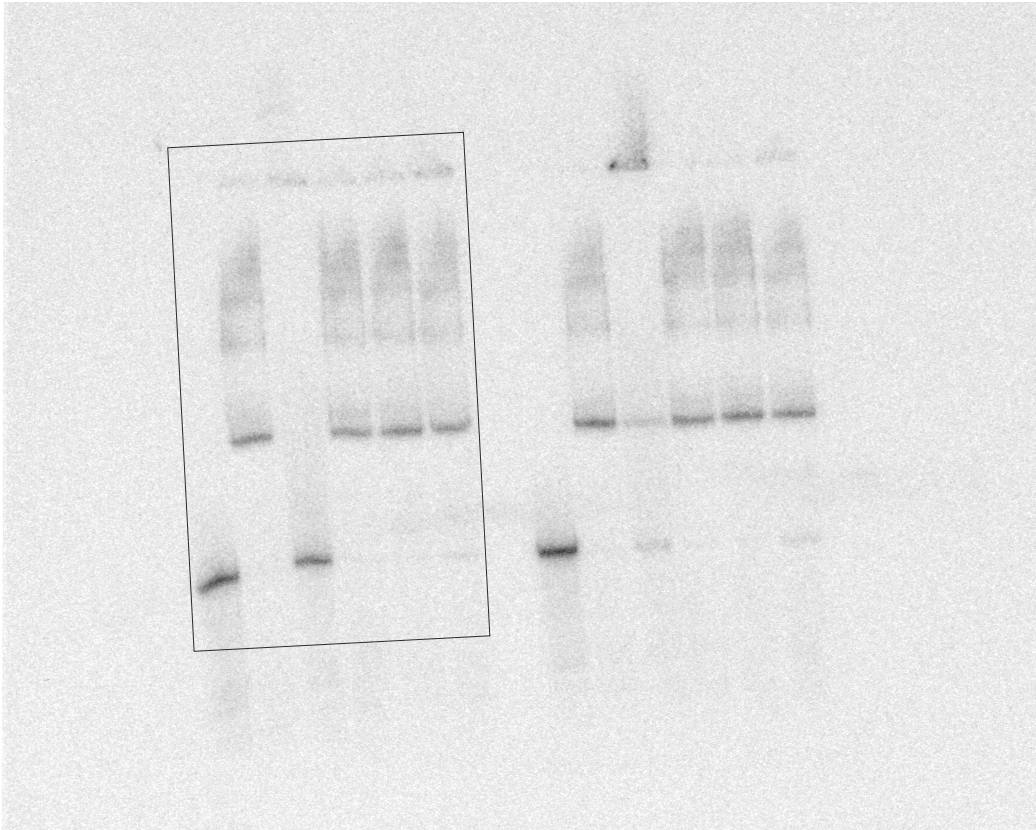

Supplement: Supplementary file 10 — Source Data for Figure 6 [file EMBJ-39-e103848-s008.zip › Source_data_Fig_6F_EMSAs.pdf]
