# Supplementary material for: Small RNA‐binding protein RapZ mediates cell envelope precursor sensing and signaling in Escherichia coli
Source: EMBO J. 2020 Feb 17;39(6):e103848. doi: 10.15252/embj.2019103848 (PMC7073468; doi:10.15252/embj.2019103848)
Supplement: Supplementary file 9 — Source Data for Figure 5 [file EMBJ-39-e103848-s007.zip › Source_data_Fig_5A_half_life_wild_type_5S_probe.pdf]

Source data_Khan_Fig5A

Fig5A, mock control, GlmY blot, 5S probe

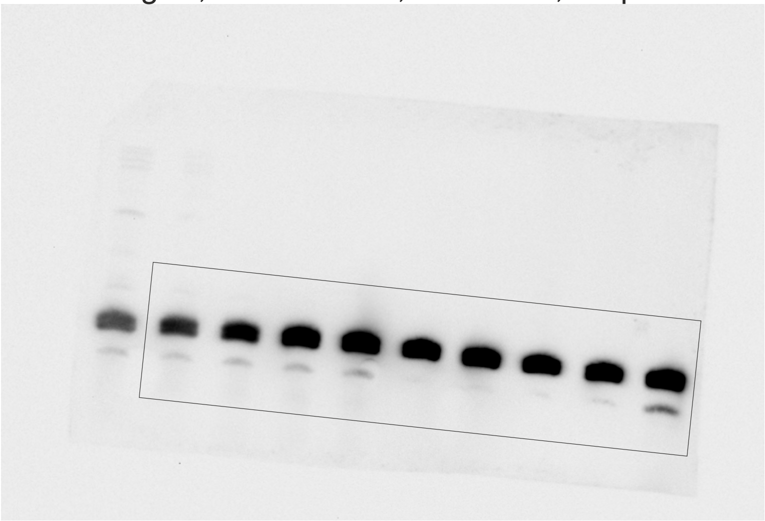

Fig5A, mock control, GlmZ blot, 5S probe

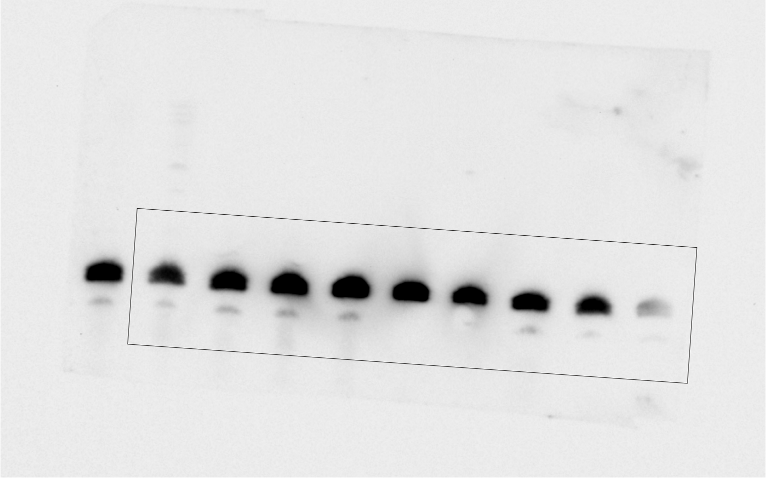

Top: Fig5A, Nva-FMDP, GlmY blot, 5S probe

Bottom: Fig5A, Nva-FMDP, GlmZ blot, 5S probe

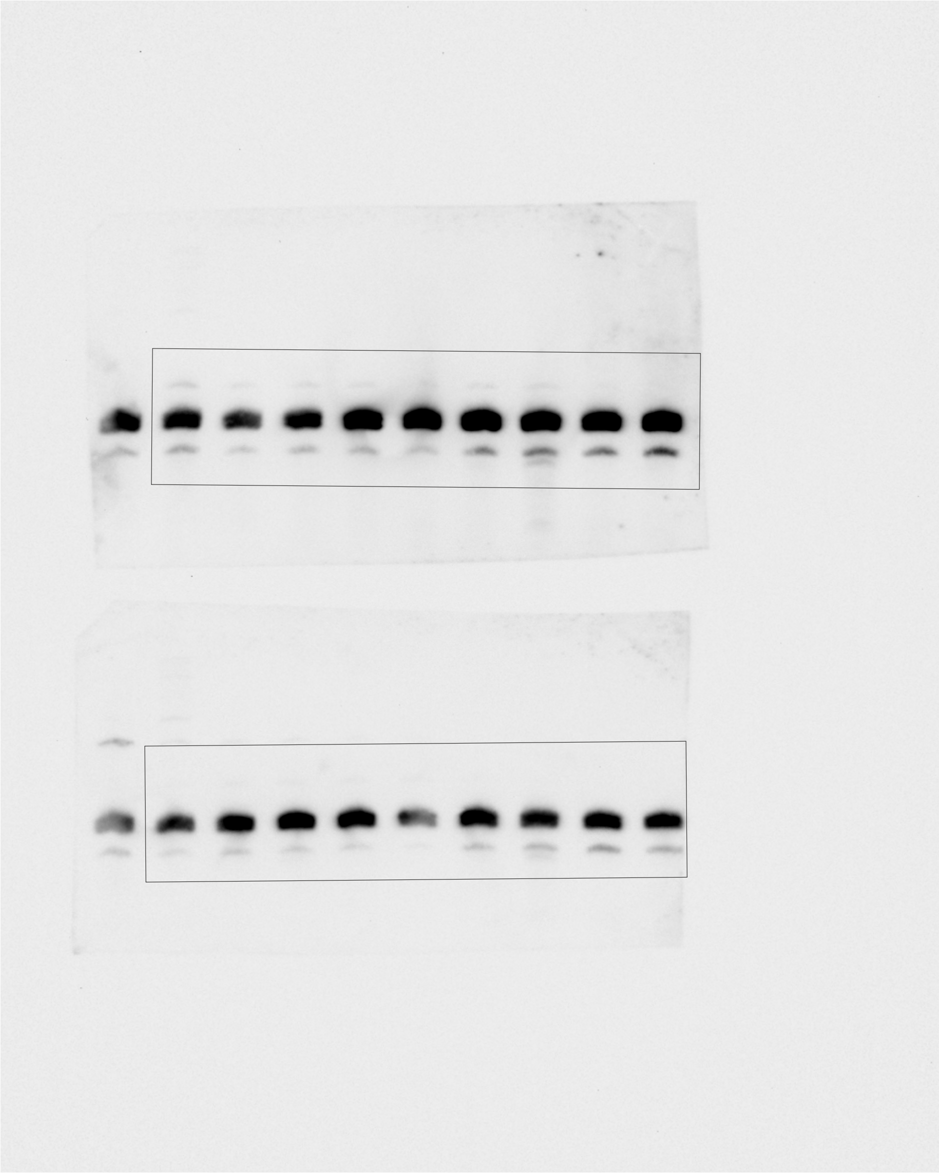

An unrelated total RNA sample was separated in lane 1, respectively, and served as a size marker
for localization of GlmY/GlmY* and GlmZ/GlmZ* in the corresponding blots (not represented in the final Figures)
