# Supplementary material for: Small RNA‐binding protein RapZ mediates cell envelope precursor sensing and signaling in Escherichia coli
Source: EMBO J. 2020 Feb 17;39(6):e103848. doi: 10.15252/embj.2019103848 (PMC7073468; doi:10.15252/embj.2019103848)
Supplement: Supplementary file 9 — Source Data for Figure 5 [file EMBJ-39-e103848-s007.zip › Source_data_Fig_5B_half_life_delta_rapZ_GlmY_GlmZ_probes.pdf]

Source data_Khan_Fig5B

Top: Fig5B, mock control, GlmY probe

Bottom: Fig5B, Nva-FMDP, GlmY probe

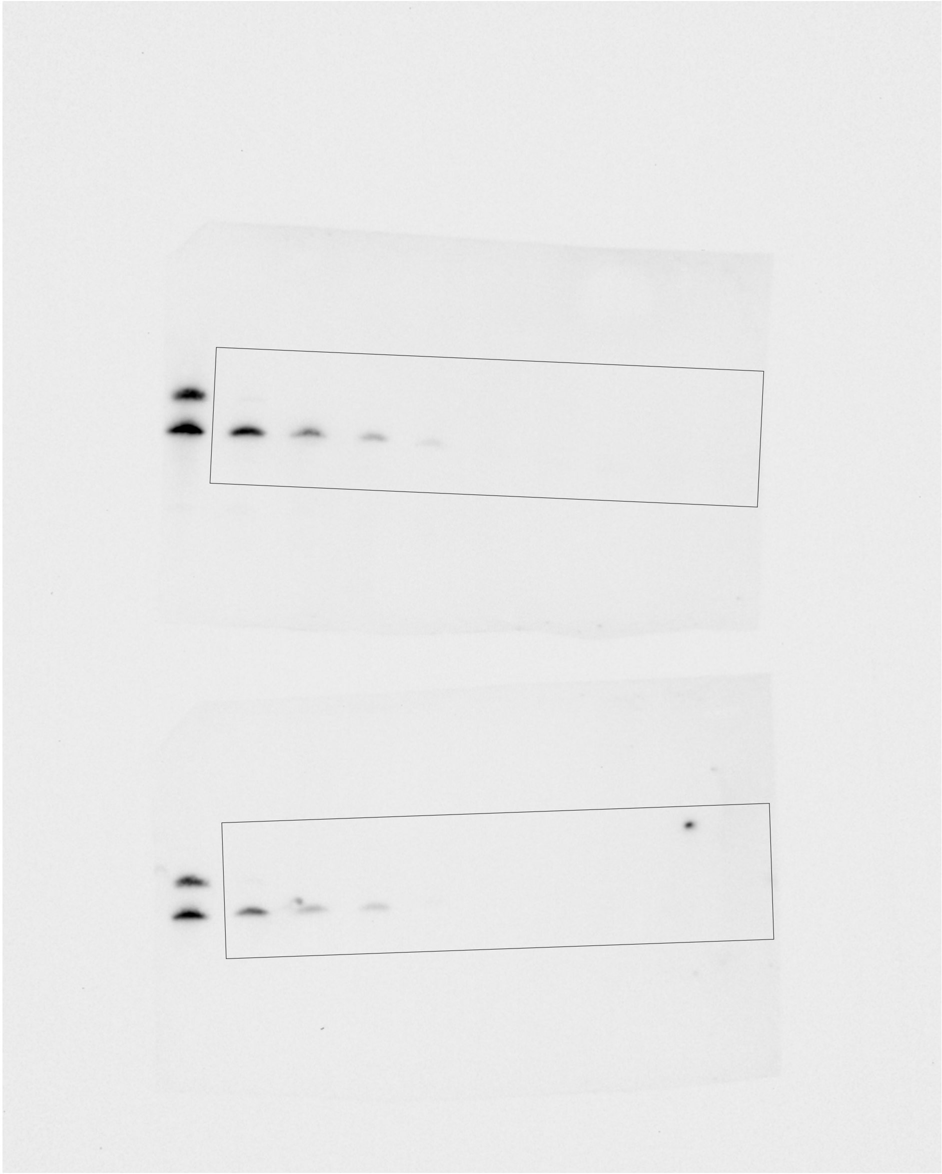

Fig5B, mock control, GlmZ probe

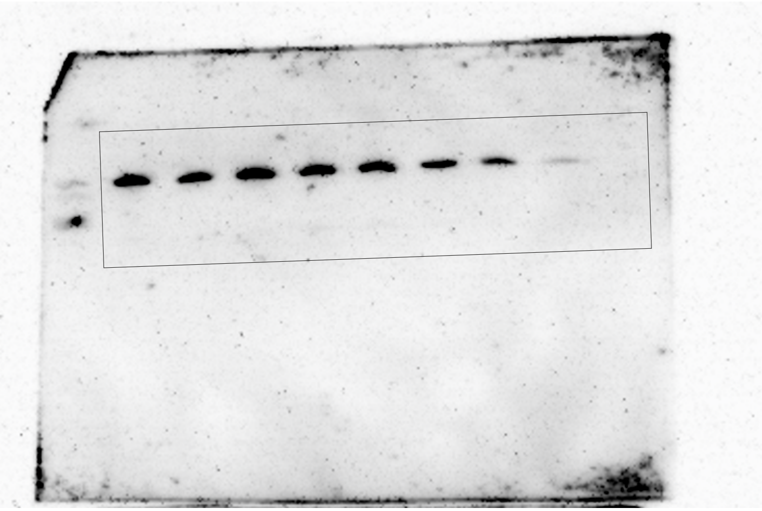

Fig5B, Nva-FMDP, GlmZ probe

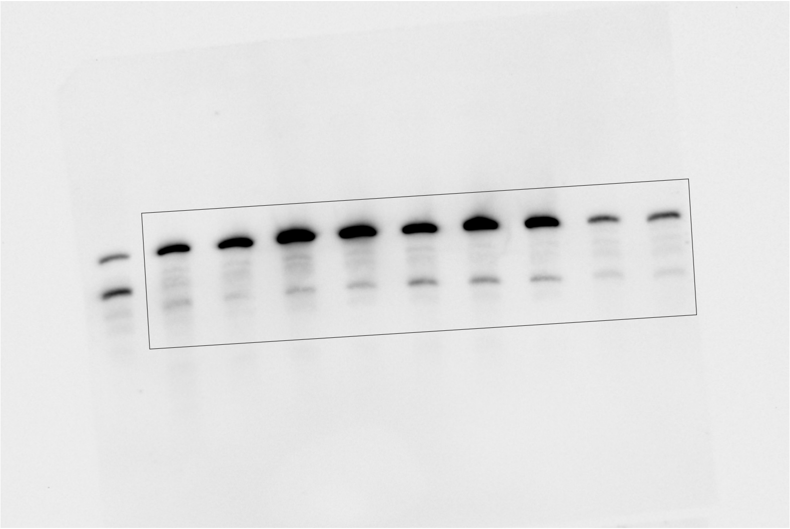

An unrelated total RNA sample was separated in lane 1, respectively, and served as a size marker
for localization of GlmY/GlmY* and GlmZ/GlmZ* (not represented in the final Figures)
