# Supplementary material for: Small RNA‐binding protein RapZ mediates cell envelope precursor sensing and signaling in Escherichia coli
Source: EMBO J. 2020 Feb 17;39(6):e103848. doi: 10.15252/embj.2019103848 (PMC7073468; doi:10.15252/embj.2019103848)
Supplement: Supplementary file 10 — Source Data for Figure 6 [file EMBJ-39-e103848-s008.zip › Source_data_Fig_6A_half_life_delta_glmS_GlmY_probe.pdf]

Source data_Khan_Fig6A

Fig6A, Δ*glmS*, +GlcN, GlmY probe

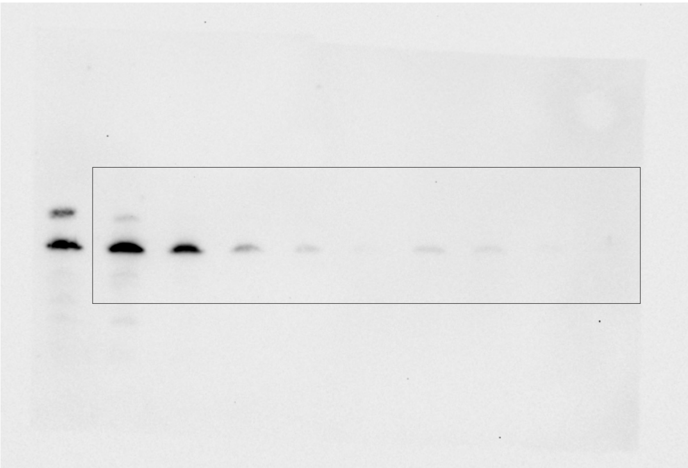

Top: Fig6A, Δ*glmS*, -GlcN, GlmY probe
Bottom: Fig6A, Δ*glmS*, -GlcN/+GlcN (8 min), GlmY probe

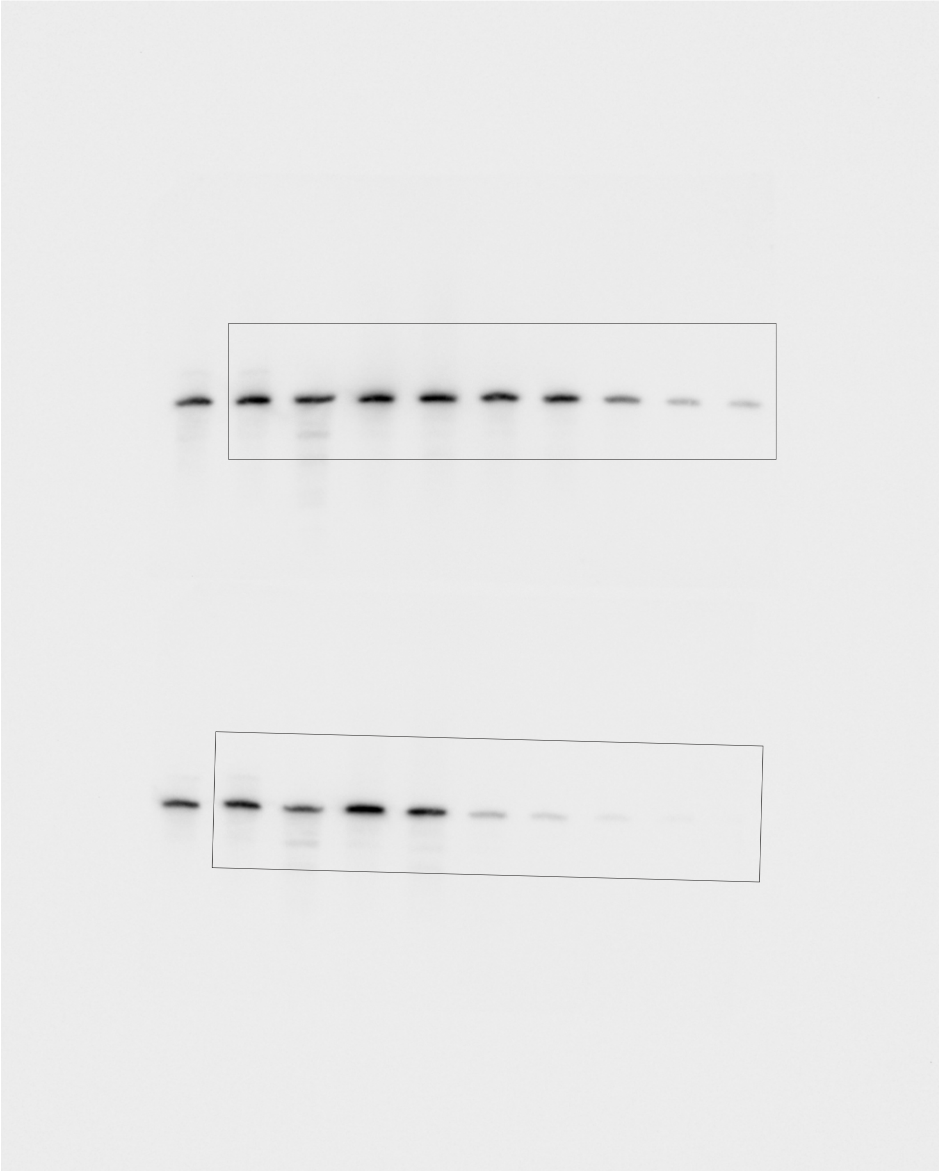

An unrelated total RNA sample was separated in lane 1, respectively, and served as a size marker for localization of GlmY/GlmY* and GlmZ/GlmZ* (not represented in the final Figures)
