# Supplementary material for: Small RNA‐binding protein RapZ mediates cell envelope precursor sensing and signaling in Escherichia coli
Source: EMBO J. 2020 Feb 17;39(6):e103848. doi: 10.15252/embj.2019103848 (PMC7073468; doi:10.15252/embj.2019103848)

Source data_Northern blots_Khan_FigEV2

FigEV2, strains Z854 (wt) and Z1082 (Δ*qseE*), GlmY probe

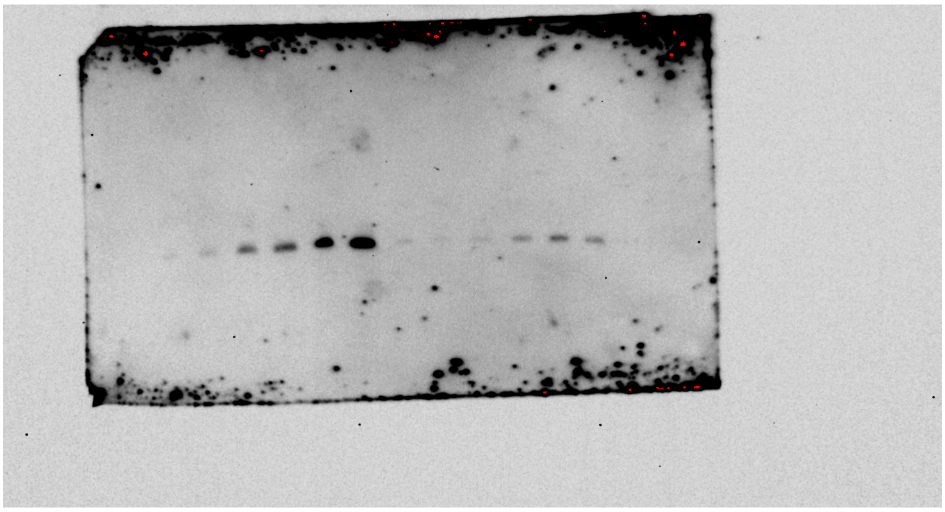

Top: FigEV2, strains Z854 (wt) and Z1081 (Δ*qseF*), GlmY probe

Bottom: FigEV2, strains Z854 (wt) and Z1083 (Δ*qseF* Δ*rapZ*), GlmY probe

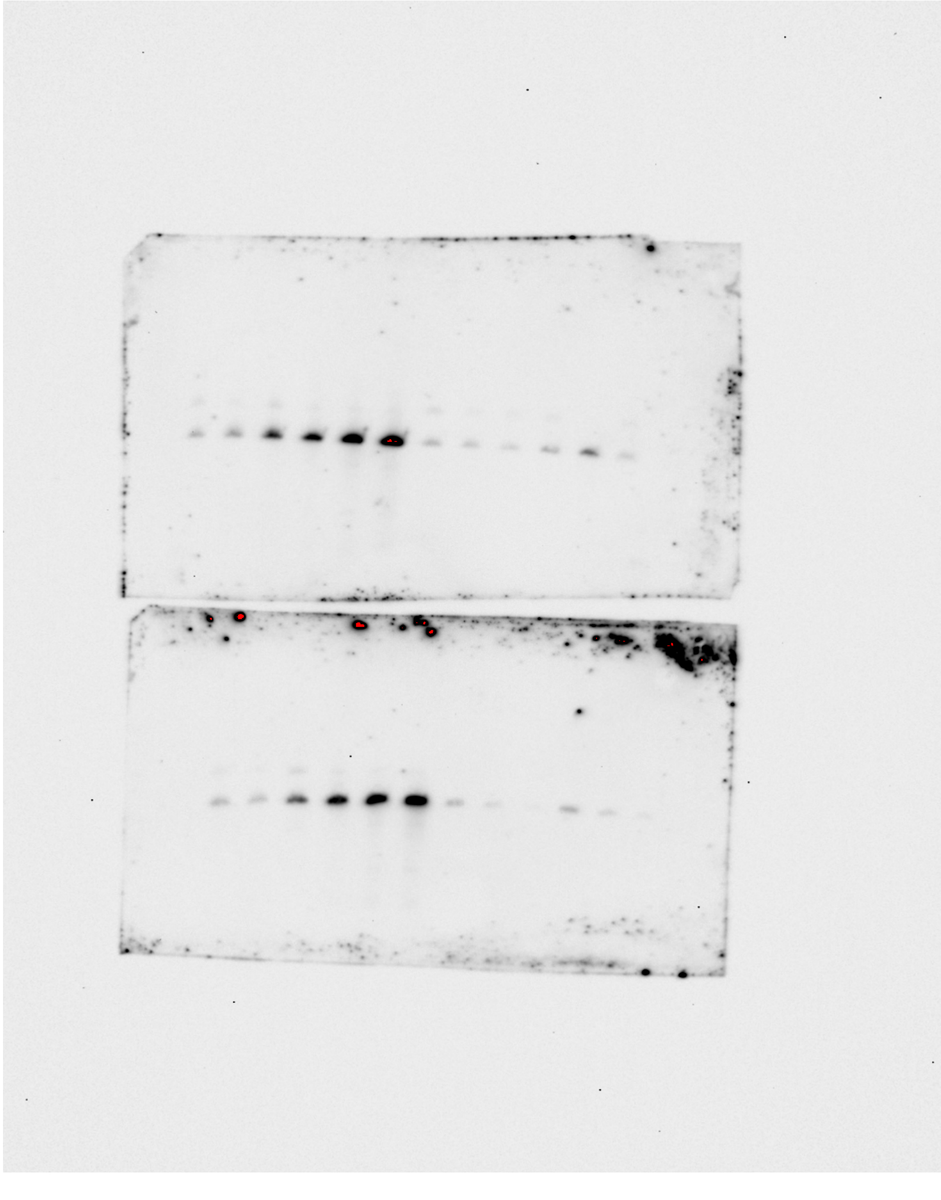

Supplement: Supplementary file 3 — Source Data for Expanded View [file EMBJ-39-e103848-s009.zip › Figure_EV2_Source_Data.zip!/Source data_FigEV2_Northern blots GlmY probe.pdf]
