# Supplementary material for: Small RNA‐binding protein RapZ mediates cell envelope precursor sensing and signaling in Escherichia coli
Source: EMBO J. 2020 Feb 17;39(6):e103848. doi: 10.15252/embj.2019103848 (PMC7073468; doi:10.15252/embj.2019103848)
Supplement: Supplementary file 3 — Source Data for Expanded View [file EMBJ-39-e103848-s009.zip › Figure_EV3_Source_Data.zip!/Source data_FigEV3_Northern blots_5S_probe.pdf]

Source data_Northern blots_Khan_FigEV3

Top: FigEV3, strain Z904 +/- GlcN, GlmY blot, 5S probe
Bottom: FigEV3, strain Z904 +/- GlcN, GlmZ blot, 5S probe

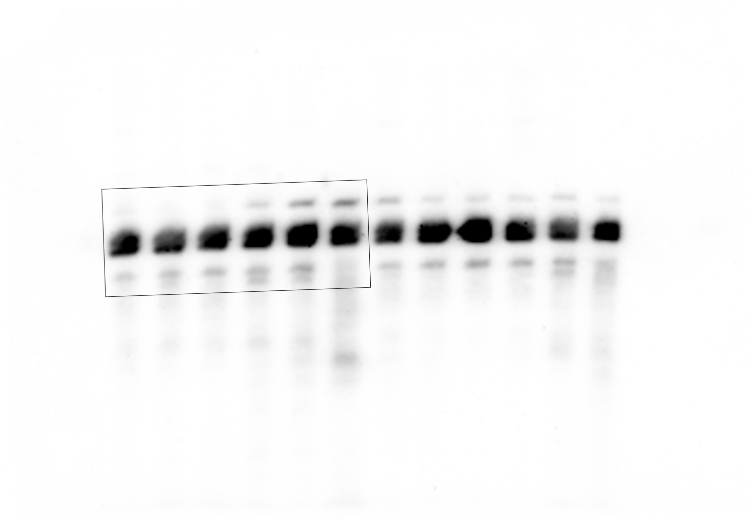

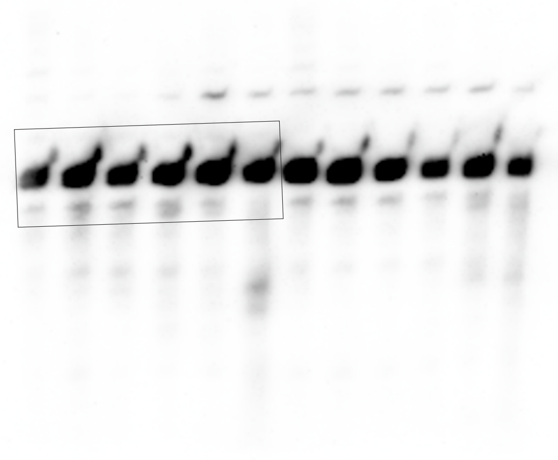

Final Figure was prepared by clipping the boxed areas
