# Supplementary material for: Small RNA‐binding protein RapZ mediates cell envelope precursor sensing and signaling in Escherichia coli
Source: EMBO J. 2020 Feb 17;39(6):e103848. doi: 10.15252/embj.2019103848 (PMC7073468; doi:10.15252/embj.2019103848)
Supplement: Supplementary file 3 — Source Data for Expanded View [file EMBJ-39-e103848-s009.zip › Figure_EV4_Source_Data.zip!/Source data_FigEV4A_half life_rapZ wt_GlmY probe.pdf]

Source data_Khan_FigEV4 A

Top: FigEV4 A, ∆*glmS,* ∆*rapZ* + *rapZ* on plasmid, +GlcN, GlmY probe

Bottom: FigEV4 A, ∆*glmS,* ∆*rapZ* + *rapZ* on plasmid, -GlcN, GlmY probe

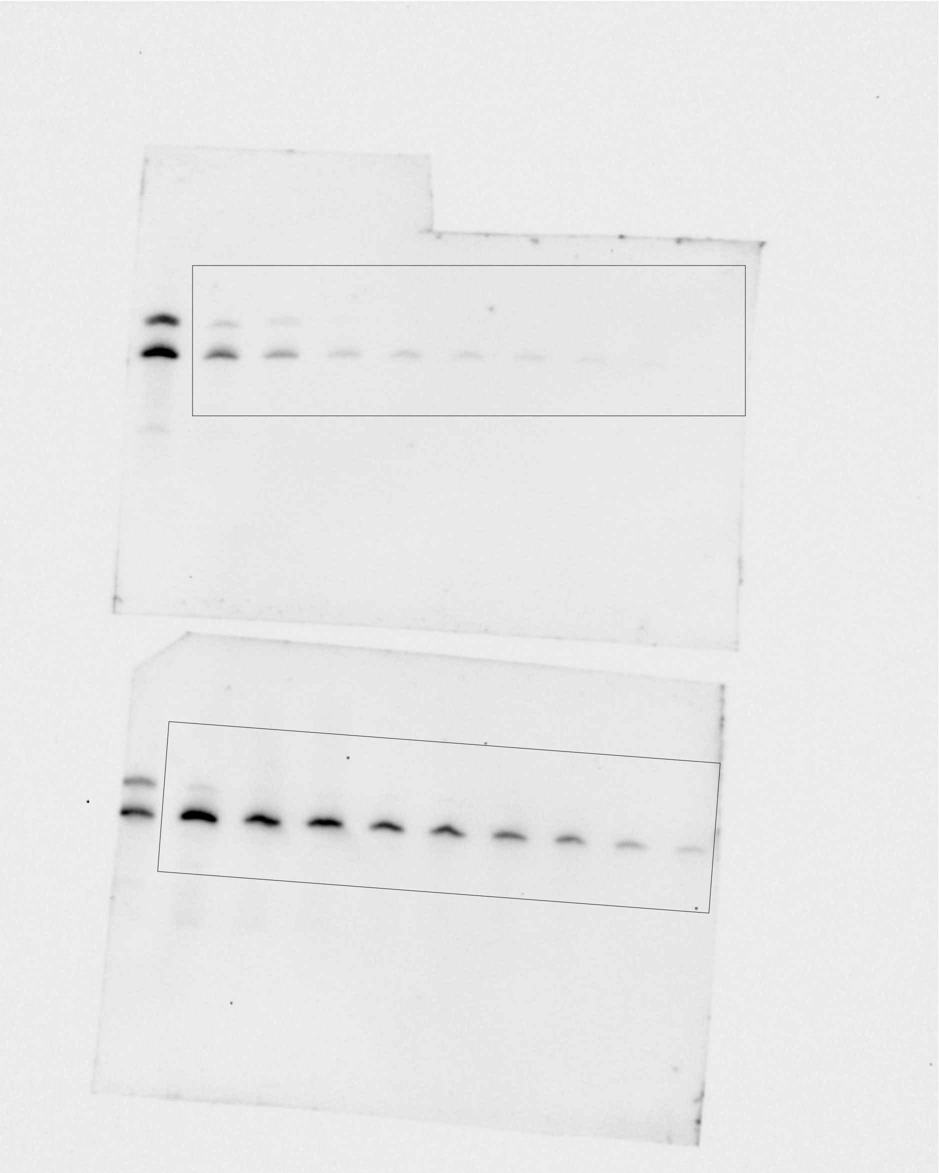

An unrelated total RNA sample was separated in lane 1, respectively, and served as a size marker for localization of GlmY/GlmY* (not represented in the final Figures)
